# Size-dependent H and H_2_ formation by infrared multiple photon dissociation spectroscopy of hydrated vanadium cations, V^+^(H_2_O)_*n*_, *n* = 3–51†‡

**DOI:** 10.1039/d2cp00833e

**Published:** 2022-04-06

**Authors:** Jakob Heller, Ethan M. Cunningham, Jessica C. Hartmann, Christian van der Linde, Milan Ončák, Martin K. Beyer

**Affiliations:** Institut für Ionenphysik und Angewandte Physik, Universität Innsbruck Technikerstraße 25 6020 Innsbruck Austria milan.oncak@uibk.ac.at martin.beyer@uibk.ac.at

## Abstract

Infrared spectra of the hydrated vanadium cation (V^+^(H_2_O)_*n*_; *n* = 3–51) were measured in the O–H stretching region employing infrared multiple photon dissociation (IRMPD) spectroscopy. Spectral fingerprints, along with size-dependent fragmentation channels, were observed and rationalized by comparing to spectra simulated using density functional theory. Photodissociation leading to water loss was found for cluster sizes *n* = 3–7, consistent with isomers featuring intact water ligands. Loss of molecular hydrogen was observed as a weak channel starting at *n* = 8, indicating the advent of inserted isomers, HVOH^+^(H_2_O)_*n*−1_. The majority of ions for *n* = 8, however, are composed of two-dimensional intact isomers, concordant with previous infrared studies on hydrated vanadium. A third channel, loss of atomic hydrogen, is observed weakly for *n* = 9–11, coinciding with the point at which the H and H_2_O calculated binding energies become energetically competitive for intact isomers. A clear and sudden spectral pattern and fragmentation channel intensity at *n* = 12 suggest a structural change to inserted isomers. The H_2_ channel intensity decreases sharply and is not observed for *n* = 20 and 25–51. IRMPD spectra for clusters sizes *n* = 15–51 are qualitatively similar indicating no significant structural changes, and are thought to be composed of inserted isomers, consistent with recent electronic spectroscopy experiments.

## Abstract 2

Infrared multiple photon dissociation spectra of V^+^(H_2_O)_*n*_ depend on experiment conditions, with strong kinetic shift effects for large clusters.

## Graphical abstract

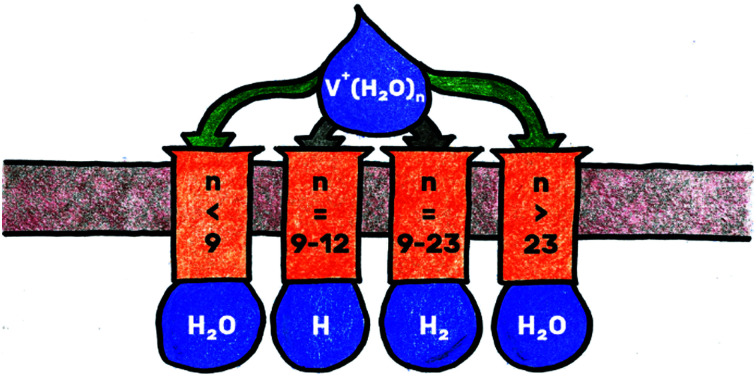

## Introduction

Hydrated ions are ubiquitous to many important biological and chemical processes.^1^ Studying gas-phase hydrated ions provides a well-defined system to investigate fundamental ion–water behavior at the molecular level.^2–6^ Important insights include solvation, hydrogen production, and corrosion mechanisms in which gas-phase studies offer the promise of bridging the gap between small clusters and bulk aqueous solution.^7–14^

Early experiments by Armentrout investigated the successive binding energies of water molecules to first row transition metals by collision induced dissociation (CID).^15^ Size-dependent fragmentation behavior of a range of hydrated metal ions has been studied by blackbody infrared radiative dissociation (BIRD),^16–19^ along with proton transfer reactions employing H_2_O/D_2_O exchange.^20,21^ BIRD experiments on V^+^(H_2_O)_*n*_ complexes (*n* = 5–30) reveal strongly size-dependent intracluster redox reactivity. Vanadium was found in oxidation state +II for cluster sizes *n* = 9–12, resulting in V(OH)^+^(H_2_O)_*n*_ ions and loss of atomic hydrogen, and the +III state for cluster sizes *n* = 9–23, leading to V(OH)_2_^+^(H_2_O)_*n*_ ions and loss of H_2_; all other cluster sizes show only water fragmentation.^19^

A wealth of spectroscopic studies have been performed on gas-phase solvated metal ions, including electronic spectroscopy,^22–34^ with many more employing infrared spectroscopy, a powerful technique used to reveal structural trends of metal–ligand clusters.^1,35–58^ The hydrated vanadium cation, however, has taken center stage and has been studied in detail by many groups using different experimental techniques. Laser ablated vanadium, when co-deposited with water molecules in an argon matrix, reacts spontaneously forming the inserted complex, HVOH.^59^ Sasaki *et al.* investigated small V^+^(H_2_O)_*n*_ complexes (*n* = 2–8) employing the inert-messenger technique, revealing a square-planar coordination of the vanadium center with four water molecules.^58^ Duncan and coworkers have performed many infrared studies on solvated vanadium complexes, the first in 2003 on V^+^(H_2_O)Ar_*n*_ and V^+^(D_2_O)Ar_*n*_ complexes in the O–H stretching region.^60^ The doubly-charged species, V^2+^(H_2_O)Ar_*n*_ (*n* = 2–7), was also studied, revealing a red-shift in absorption frequencies compared to V^+^(H_2_O), and a coordination of six to V^2+^; one water molecule and five argon atoms.^47^ Later, the Duncan group also recorded the infrared spectra of V^+^(H_2_O) with different rare-gas messenger atoms, reporting evidence of *ortho*-para conversion on the water molecule.^61^ More recently, Duncan and coworkers extended these infrared studies to larger clusters, investigating the microsolvation of V^+^ up to 30 water molecules.^62^ Small clusters were probed using argon tagging, while larger clusters, *n* > 7, were studied *via* the photodissociation of intact water molecules. The spectra, when coupled with simulated spectra from density functional theory, present a coordination number of four, concordant with a square-planar structure. Clusters up to *n* = 8 remain two-dimensional, however in larger clusters hydrogen bonding networks dominate, forming three-dimensional structures.

Recently, we performed electronic photodissociation experiments in the ultraviolet/visible (UV/Vis) region on V^+^(H_2_O)_*n*_ ions (*n* = 1–41).^63^ Clusters up to 12 water molecules exhibit intense 3d–4p transitions which red-shift until the first solvation sphere is reached at *n* = 4. At 9 water molecules, the intense absorption bands begin to disappear as V^+^ inserts into the O–H bond of a water molecule, forming [HVOH(H_2_O)_*n*−1_]^+^ whereby the oxidation state of V changes from +I to +III. Loss of water molecules, along with competing loss of atomic and molecular hydrogen, is observed for *n* ≤ 12.

To confirm the presence of specific structural isomers inferred from the UV/Vis spectra, this study utilizes infrared multiple photon dissociation (IRMPD) spectroscopy in the O–H stretching region investigating the solvation evolution of V^+^(H_2_O)_*n*_ ions up to *n* = 51. The goal is to establish the presence of inserted isomers at particular clusters sizes and any possible infrared-driven H and H_2_ loss. The infrared-driven elimination of molecular hydrogen was recently observed for particular HAlOH^+^(H_2_O)_*n*−1_ clusters, *n* = 9–14, probed in the 1400–2250 cm^−1^ region.^64^ Presence of a hydride was found to be crucial in the elimination of molecular hydrogen, and found to occur only for cluster sizes *n* = 12, 13 and 14.

## Experimental and computational methods

The experiments are performed on a 4.7 Tesla Fourier-Transform Ion Cyclotron Resonance Mass Spectrometer (FT-ICR MS) Bruker Spectrospin CMS47X.^65–68^ The mass spectrometer is equipped with a Bruker infinity cell^69^ along with a laser vaporization source,^70,71^ where a solid disk of vanadium is vaporized by a frequency doubled Litron Nano S 60-30 Nd:YAG laser (532 nm, 5 mJ per pulse, 30 Hz). The plasma containing V^+^ is entrained in a pulse of helium seeded with water vapor created *via* a homebuilt piezoelectric valve following the design of Proch and Trickl.^72^ The ensuing pulse is cooled *via* supersonic expansion, whereby hydrated vanadium complexes, V^+^(H_2_O)_*n*_, are created. The clusters are transferred into the ICR cell where they are stored and mass-selected within the 4.7 T magnetic field^73^ under ultra-high vacuum conditions (*p* ≈ 5 × 10^−10^ mbar).

The mass-selected ions of interest are irradiated with infrared light provided by an EKPLSA NT277 Optical Parametric Oscillator (OPO) covering the range from 2240–4000 cm^−1^. Typical irradiation times are 0.1–0.2 s at 1000 Hz repetition rate. The normalized IRMPD yield is calculated from the precursor ion and fragment ion intensities^74^ and scaled with the laser power. Ion intensities are normalized to 100% precursor ion intensity. The fragment intensities are then BIRD corrected by subtracting the fragment intensities from a control experiment without laser irradiation. IRMPD yields are calculated from the corrected ion intensities, normalized by the wavelength-specific laser power and re-normalized for display as described before.^75^

The laser power is measured after every mass spectrum to account for any fluctuations. The laser power drops in the range of 3500–3520 cm^−1^. Due to the complex kinetics of the IRMPD process, this may lead to small artefacts in the IRMPD yield at these wavelengths even after power correction.

Especially for larger clusters, dissociation due to BIRD from the cell walls can occur. To prevent this, the ICR cell is surrounded by a copper jacket, whereby the ions can be cooled with liquid nitrogen to a temperature of *ca.* 90 K,^16,76^ minimizing the effects of BIRD.^77–82^ The remaining BIRD fragmentation was taken into account by subtraction of the measured fragment ion intensities with a reference mass spectrum where the ions were trapped without irradiation from the OPO.

To complement the experimental photodissociation spectra, density functional theory (DFT) calculations were performed generating structures of V^+^(H_2_O)_*n*_ at the B3LYP/aug-cc-pVDZ level of theory. Simulated infrared bands are scaled by a factor of 0.96 to compensate for anharmonicity and method deficiency, and spectra are generated by implementing Gaussian functions to band positions, each with a full-width-half-maximum (FWHM) of 20 cm^−1^. All calculations were performed using the Gaussian 16 package.^83^ Relative energies of isomers are given in kJ mol^−1^, inclusive of zero-point energy. All isomers of the intact V^+^(H_2_O)_*n*_ structure considered in this study are present in the quintet ground state, with other multiplicities lying higher in energy. The inserted HVOH^+^(H_2_O)_*n*_ structures were found in triplet multiplicity, and are more stable than their singlet and quintet analogues. Wave function stabilization was performed prior to each structure optimization.

## Results and discussion

**Fig. 1:**
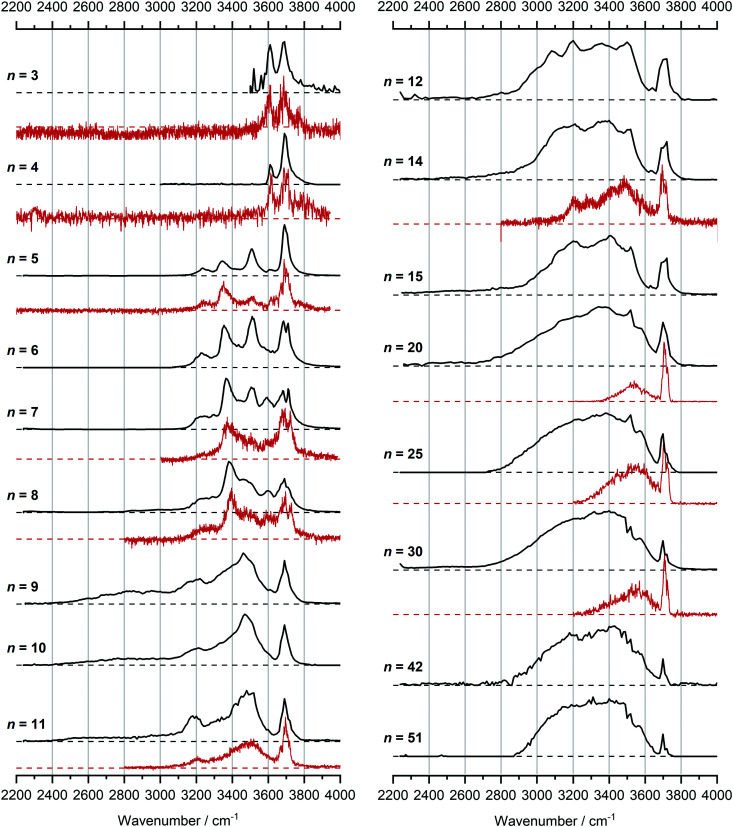
Infrared multiple photon dissociation spectra of the V^+^(H_2_O)_*n*_ complexes (*n* = 3–12, 14, 15, 20, 25, 30, 42 and 51) presented in black. For comparison, infrared spectra from Duncan and coworkers are also presented in red for cluster sizes *n* = 3–5, 7, 8, 11, 14, 20, 25 and 30.^62^

**Fig. 2:**
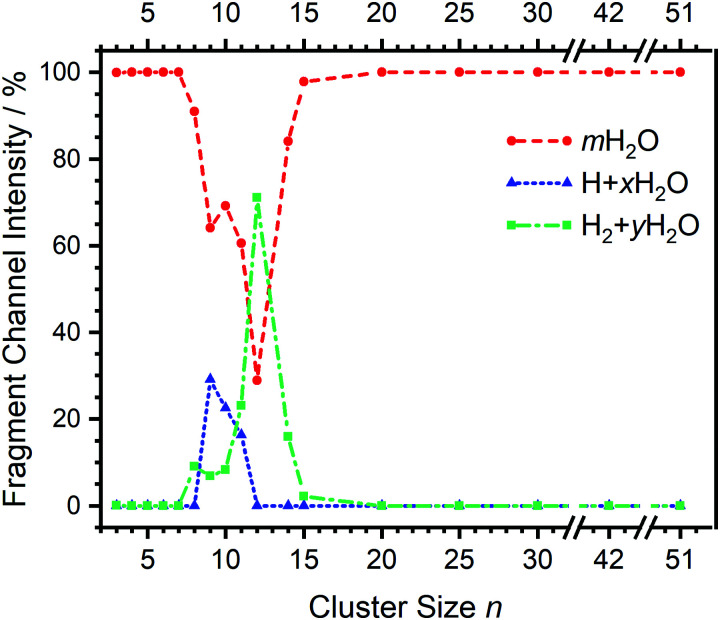
Fragmentation channel intensities for all investigated clusters. The points are connected with lines to guide the eye.

In Fig. 1, we compare the measured IRMPD spectra of V^+^(H_2_O)_*n*_, *n* = 3–51, Fig. 2 includes fragmentation intensities of the observed dissociation channels. Infrared spectra of Ar and N_2_ tagged as well as untagged V^+^(H_2_O)_*n*_ have been published previously in two separate studies by the groups of Duncan and Ohashi, and the original spectra from the Duncan group are included in Fig. 1.^58,62^ Our photodissociation spectra up to *n* = 7 are concordant with these previous studies. Since no tagging technique is used in this work, investigations of smaller systems (*n* ≤ 2) are not possible due to inefficient elimination of H_2_O. For cluster sizes *n* ≥ 8, differences start to emerge between the IRMPD spectra measured in the present work (black spectra) and those recorded by Duncan and coworkers (red spectra).^62^ Most notably, the broad and weak red-shifted feature at 2500–3000 cm^−1^ seen in our IRMPD spectrum for *n* = 11 is not present in the *n* = 11 spectrum recorded by Duncan. Going larger to *n* = 30, the strongest feature is a broad absorption at 2800–3650 cm^−1^, with weaker, “free” O–H bands at 3700 and 3720 cm^−1^. Duncan's spectrum shows the opposite, the “free” O–H bands are the most intense, while the absorption at 3200–3650 cm^−1^ is weaker and spectrally narrower. These spectral differences can be attributed to the disparities in the two experimental methods employed. In particular, a time-of-flight mass spectrometry instrument is used in the study by Duncan, employing single-photon infrared photon dissociation (IRPD) methods. Upon increasing the number of water molecules, the heat capacity of the cluster increases, which means water evaporation from the cluster becomes more demanding energetically. Thus, under a single-photon regime, the photofragmentation rate decreases upon increasing cluster size. In contrast, clusters in the present study are trapped in the center of an ICR cell, whereby infrared multiple photon dissociation (IRMPD) spectroscopy is employed. Therefore, any increases in heat capacity can be readily overcome by introducing more photons, causing significant photodissociation.

**Fig. 3:**
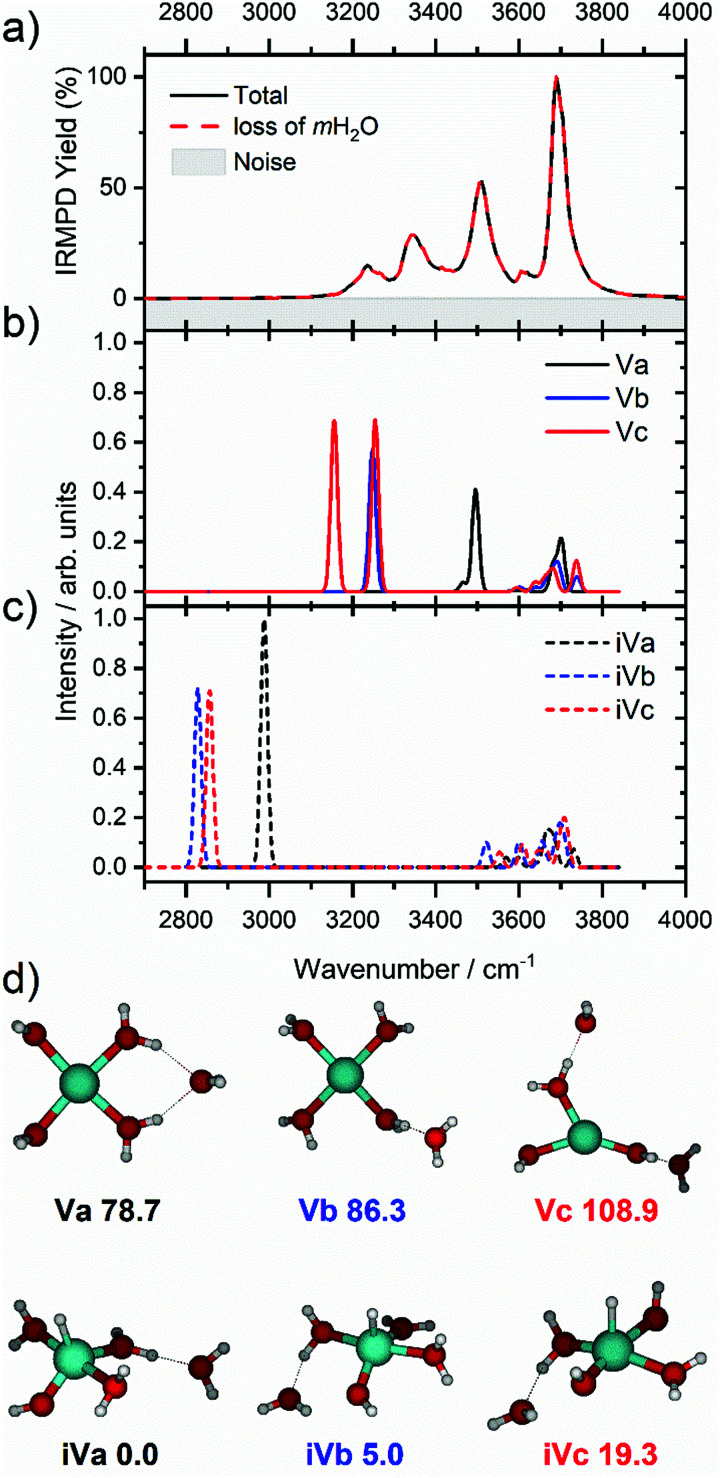
(a) Infrared multiple photon dissociation spectrum of the V^+^(H_2_O)_5_ complex along with the simulated spectra of low-lying (b) intact (Va–Vc) and (c) inserted (iVa–iVc) isomers calculated at the B3LYP/aug-cc-pVDZ level of theory. (d) Structural isomers of intact (Va–Vc) and inserted isomers (iVa–iVc) with relative energies given in kJ mol^−1^ inclusive of zero-point energy. In each case the observed photodissociation events in a) are due to the sequential loss of intact water molecules, *m*H_2_O.

We start our discussion with the smallest clusters, *n* = 3–7, for which we see structured spectra with clearly defined bands. The only observed dissociation channel is water evaporation. In Fig. 3, we analyze the experimental as well as theoretical spectra of V^+^(H_2_O)_5_ as an example (spectra of other small clusters are analyzed in Fig. S1–S3, ESI‡). The experimental spectrum is in excellent agreement with the untagged spectra measured by Duncan, as well as the spectra recorded by Ohashi.^58,62^ As shown in Fig. 3, all three low-lying isomers of the intact V^+^(H_2_O)_5_ present a good agreement with the bands in the observed spectrum, possessing both the four- (Va, Vb) and three-coordinate (Vc) vanadium cation. The bands observed at 3510 and 3690 cm^−1^ can be explained by isomer Va, consistent with Duncan's results of the V^+^(H_2_O)_5_·Ar cluster.^62^ The feature at 3345 cm^−1^ can be ascribed to the single-acceptor water molecule of isomer Vb, and both features at 3345 and 3230 cm^−1^ are in reasonable agreement with the two single-acceptor water molecules in Vc. In principle, the feature at 3230 cm^−1^ can also be assigned to the bending overtone of isomer Va, as proposed by Ohashi and coworkers.^58^ However, our anharmonic frequency analysis shows that, while there are indeed bending overtones in the 3100–3200 cm^−1^ region, their intensity is two orders of magnitude lower compared to the intensity of the most intense O–H stretch. The four bands present in the observed IRMPD spectrum can be thus ascribed to contributions from only intact isomers.

The inserted species, HVOH^+^(H_2_O)_4_ (iVa–iVc), are considerably more stable than the intact clusters (Fig. 3d). However, they show intense red-shifted bands below 3000 cm^−1^ which are not observed experimentally. Thus, the barrier to insertion must be too high to attain at these temperatures for this cluster size. This is also consistent with previous BIRD investigations on V^+^(H_2_O)_5_, which presented only dissociation of water molecules.^19^

**Fig. 4:**
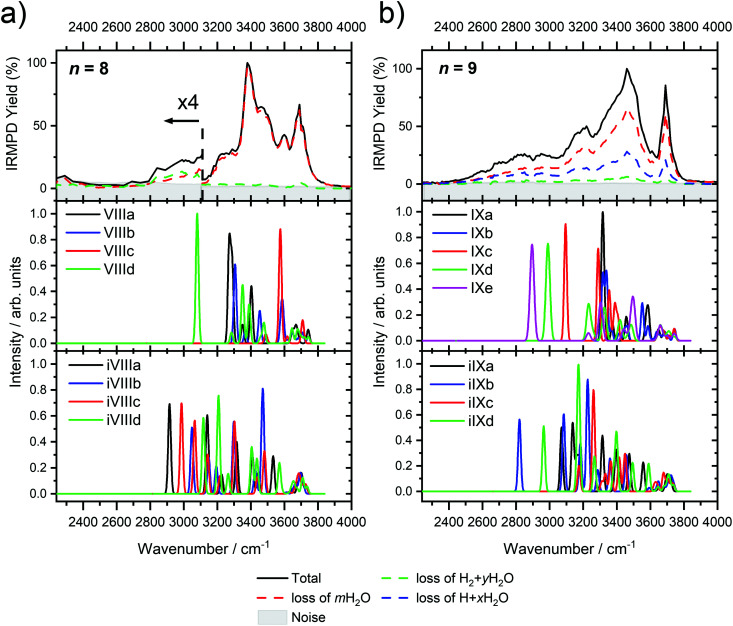
Infrared multiple photon dissociation spectra of (a) V^+^(H_2_O)_8_ and (b) V^+^(H_2_O)_9_ along with the simulated photodissociation spectra of low-lying intact and inserted isomers calculated at the B3LYP/aug-cc-pVDZ level of theory. In each case, photodissociation events are due to sequential loss of either (i) loss of intact water molecules *m*H_2_O, (ii) loss of H + *x*H_2_O, and (iii) loss of H_2_ + *y*H_2_O, represented as dashed red, blue, and green lines, respectively.

Starting with *n* = 8, H_2_ evaporation appears as a new reaction channel, although sequential loss of water molecules is still the main channel (Fig. 2), consistent with previous BIRD studies of V^+^(H_2_O)_8_.^19^ The number, and composition, of ligands lost during the IRMPD process for each cluster size is given in the ESI‡ (see Table S1). In each case, *m* denotes the sequential number of water molecules, *x* denotes the number of waters following H dissociation, and *y* the number of waters following H_2_ dissociation. Fig. 4a presents the IRMPD spectrum measured for V^+^(H_2_O)_8_, showing a qualitatively similar IRMPD spectrum as observed for *n* = 5–7. As discussed by Duncan,^62^ upon increasing cluster size the structural evolution of V^+^(H_2_O)_*n*_ clusters retains a quasi-planar, square structural motif up to *n* = 8, presenting a metal coordination number of four. The two-dimensional structure is broken with inserted isomers. Different to the experimental spectrum recorded by Duncan, the spectrum in Fig. 4a shows the presence of a weak feature between 2790–3110 cm^−1^.

**Fig. 5:**
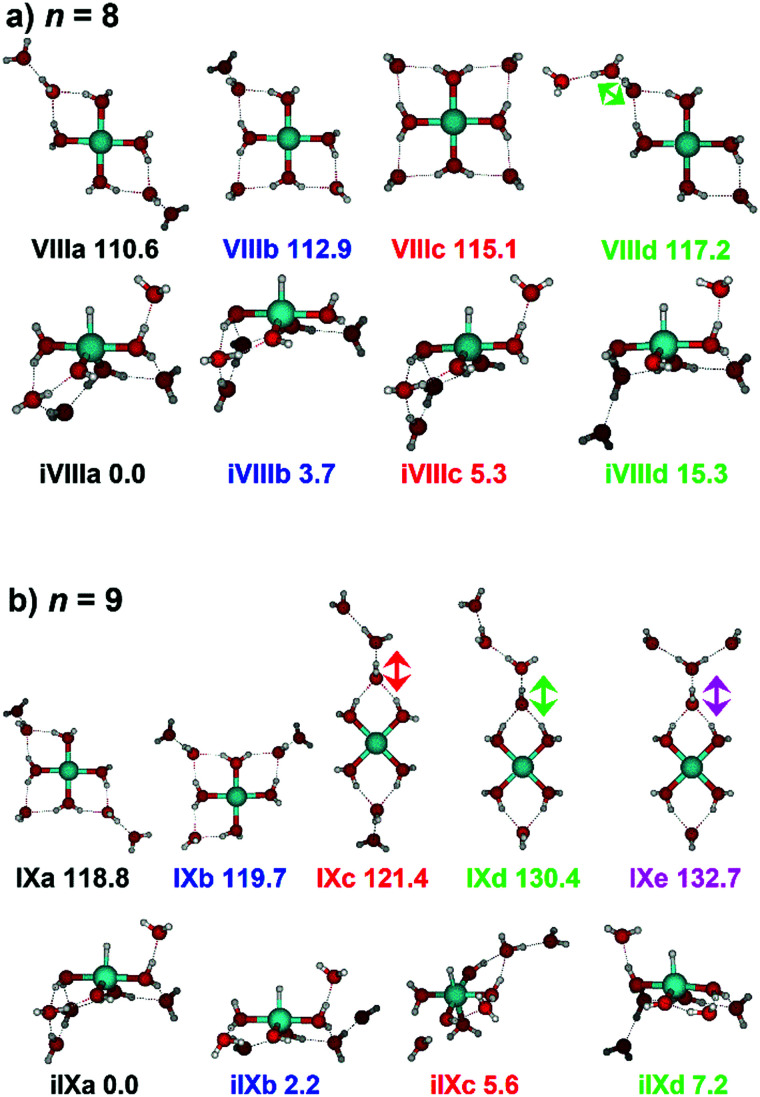
Calculated intact and inserted isomers of (a) V^+^(H_2_O)_8_ and (b) V^+^(H_2_O)_9_. All isomers were calculated at the B3LYP/aug-cc-pVDZ level of theory with relative energies given in kJ mol^−1^ inclusive of zero-point energy. Arrows indicate the O–H stretch of the most red-shifted band, in each case a water molecule in a double acceptor binding motif in the second solvation sphere.

IR spectra provided by quantum chemical calculations offer two possible explanations for the low-intensity tail of the spectrum. The band could correspond to inserted isomers (iVIIIa–iVIIId, see Fig. 5a) as these spectra present red-shifted bands in this region. However, the intact isomer VIIId also presents a band in this region at 3078 cm^−1^. This vibration is represented by a green arrow in Fig. 5a, showing the O–H stretch in a second solvation sphere water molecule, in a double-acceptor site bound to another third solvation sphere water molecule. The observed red-shift signifies significant weakening of the O–H bond, and could indicate the onset of H atom transfer from the second sphere water molecule, forming H_3_O^+^ in the third sphere. Isomer VIIId is also only 7 kJ mol^−1^ less stable than the most stable intact isomer found, VIIIa.

The discrepancy between the spectrum recorded in the present study and that by Duncan could be rationalized by temperature effects; for clusters thermalized in an ICR cell, warmer clusters are present, where entropic effects dominate enthalpic effects. More entropic water binding motifs with red-shifted bands, such as single-acceptor sites observed in VIIId, are present. This effect has been observed before in our own infrared studies on hydrated zinc cations^55,84^ and in IR studies on M^+^(H_2_O)_*n*_ complexes elsewhere.^85,86^

The main contribution to the red-shifted feature between 2790–3110 cm^−1^ is the H_2_ + H_2_O fragmentation channel (dashed green spectrum), and is slightly more intense than the intact water loss channel (dashed red spectrum). Based on previously calculated H_2_ loss mechanisms of Al^+^(H_2_O)_20_, pathways involving inserted isomers are more energetically favorable.^87,88^ Briefly, a proton moves from a first to a second shell water molecule leading to H_3_O^+^. Through consecutive proton transfer through a “water wire”, the proton reaches a site near the hydride statistically. When the proton is pointing towards the hydride at the metal center, the hydride and the proton can recombine leading to the release of an H_2_ molecule. The energy released from this recombination step leads to evaporation of multiple H_2_O molecules, see calculated reaction energies in Table 1. Thus, based on the weakly observed H_2_ + H_2_O fragment channel, there is a small contribution from inserted isomers. However, the majority of the ions present in the experiment are intact structures, V^+^(H_2_O)_8_, as can be deduced from the low-intensity red tail of the spectrum. The energetically less stable intact isomers are generated in the source chamber, while the inserted isomers, HVOH^+^(H_2_O)_7_, are generated *via* insertion and consecutive water fragmentation of larger clusters. A possible 2H + H_2_O dissociation channel can be discarded as it lies by 4.48 eV (H_2_ dissociation energy) higher in energy and is unlikely to contribute.

**Table 1:** Calculated insertion energies (*E*_ins_) and dissociation energies (all in kJ mol^−1^) for intact and inserted isomers of V^+^(H_2_O)_*n*_ stoichiometry. Calculated at the B3LYP/aug-cc-pVDZ level of theory

*n*	*E*_ins_	Intact (V^+^(H_2_O)_*n*_)			Inserted (HVOH^+^(H_2_O)_*n*−1_)		
		H_2_O	H	H_2_	H_2_O	H	H_2_
5	−79	50	116	−142	63	195	−63
6	−90	42	88	−156	54	178	−66
7	−103	39	63	−174	52	165	−71
8	−111	40	45	−185	48	156	−74
9	−119	35	34	−196	43	153	−77

**Fig. 6:**
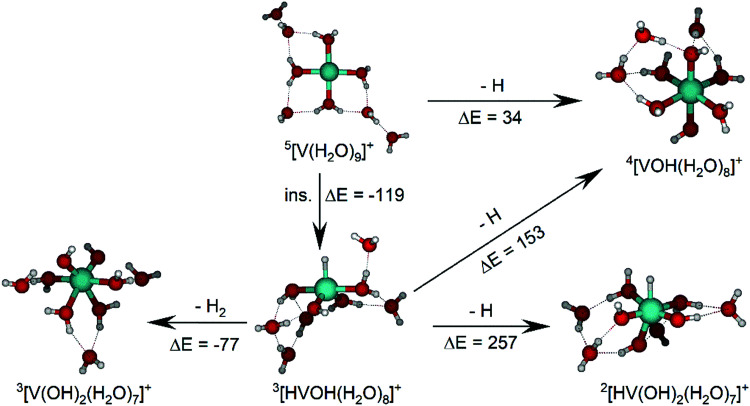
Calculated intact and inserted isomers of V^+^(H_2_O)_9_, along with insertion energy (ins.), molecular hydrogen and atomic hydrogen dissociation energy pathways. All isomers and energy pathways were calculated at the B3LYP/aug-cc-pVDZ level of theory with relative energies given in kJ mol^−1^ inclusive of zero-point energy.

The IRMPD spectrum of *n* = 9 is presented in Fig. 4b, showing that the dissociation of water molecules is the dominant fragmentation channel, followed by H + H_2_O, then the H_2_ + H_2_O channel. Similar fragmentation channels were observed in BIRD experiments, suggesting the same reactions take place with different heating mechanisms.^19^ Based on the discussion above, we can attribute H_2_ loss to the presence of inserted isomers, HVOH^+^(H_2_O)_8_. The H loss, on the other hand, can be assigned only to intact isomers as it is energetically too demanding for the inserted species (153 kJ mol^−1^, Table 1). In addition, the energy required to dissociate an H atom from an intact cluster (34 kJ mol^−1^) becomes isoenergetic with H_2_O evaporation for *n* = 9 (Table 1). The H + H_2_O dissociation channel, along with the water dissociation channel, is thus assigned to intact isomers, V^+^(H_2_O)_9_. In other words, the thermochemical considerations point at the co-existence of both cluster types. The intact and inserted isomers of V^+^(H_2_O)_9_, along with the atomic and molecular hydrogen dissociation energy pathways, are presented in Fig. 6. The lowest energy pathway starts with an intact isomer ^5^[V(H_2_O)_9_]^+^, with H lost from a water molecule (*i.e.* without insertion, resulting in product ^4^[VOH(H_2_O)_8_]^+^). The insertion step is exothermic by 119 kJ mol^−1^, which would certainly result in evaporation of multiple water molecules. Nevertheless, atomic H loss from the inserted species ^3^[HVOH(H_2_O)_8_]^+^ requires 257 kJ mol^−1^, resulting in the inserted product ^2^[HV(OH)_2_(H_2_O)_7_]^+^. Atomic H loss from the inserted species ^3^[HVOH(H_2_O)_8_]^+^, resulting in ^4^[VOH(H_2_O)_8_]^+^, is also energetically demanding, calculated as 153 kJ mol^−1^. Based on these energies, the photodissociation spectrum of H + H_2_O in Fig. 4b originates from intact isomers.

Similar to *n* = 8, the spectrum of *n* = 9 also shows a broad structureless absorption at 2410–3640 cm^−1^, the “free” O–H band at 3690 cm^−1^ and a broad red-shifted feature at 2410–3000 cm^−1^ which gains intensity and an increased spectral width for *n* = 9. Fig. 4b also shows the simulated spectra of calculated intact isomers, IXa–IXe, and inserted isomers, iIXa–iIXd, the structures of which are shown in Fig. 5b. The broad absorption at 2410–3640 cm^−1^ is the hydrogen bonding region composed of many different O–H environments with varying degrees of red-shift, with both intact and inserted isomers presenting agreement. The “free” O–H band at 3690 cm^−1^ also shows agreement with both isomer classes.

The red-shifted feature at 2410–3000 cm^−1^ with all three reaction channels present provides particular insight into the cluster structure. As discussed above, we attribute the feature to the presence of both inserted isomers iIXb and iIXd (showing bands at 2820 and 2965 cm^−1^, respectively) and the intact isomers, namely IXc, IXd, and IXe with bands at 3095, 2990, and 2895 cm^−1^, respectively. The O–H stretch of the latter bands are shown as arrows in Fig. 5b, in each case the O–H bond of a second-sphere water molecule in a double-acceptor motif, bound to a third-sphere water molecule. Isomer IXe shows the most red-shifted band, which is caused by two fourth-sphere water molecules bound to the third-sphere water molecule acting on the second-sphere water. Induced polarisation effects onto the second-sphere water molecule from the metal center, and from the third-sphere water molecule, remove electron density from the bonding orbitals in the O–H bond, which weaken the bond causing the red-shift.

We conclude that the majority of the ions for *n* = 9 are composed of intact isomers, however *n* = 9 represents the cluster size at which the abundance of inserted isomers starts to increase. Our conclusions are consistent with our previous investigation of V^+^(H_2_O)_*n*_ in the ultraviolet/visible range, with a decrease in photodissociation cross section starting at *n* ≈ 9, decreasing further upon increasing water until *n* ≥ 15 where no photoinduced fragmentation was observed, indicating an oxidation state change from V(i) to V(iii).^63^

**Fig. 7:**
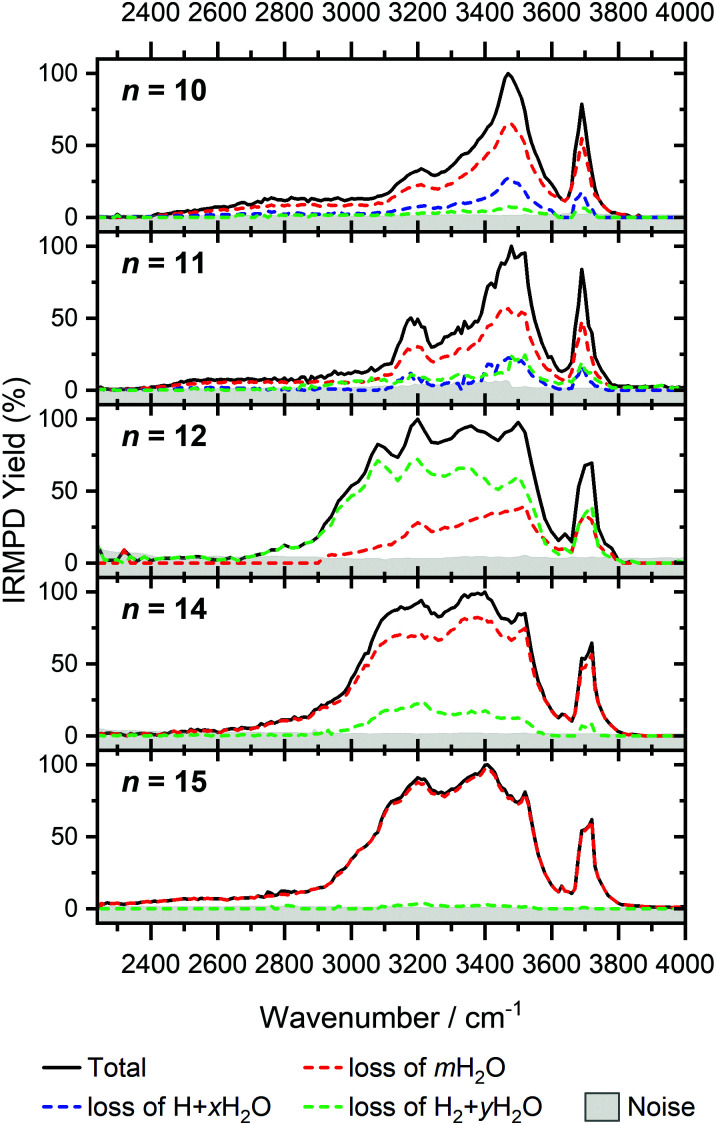
Infrared multiple photon dissociation spectra of V^+^(H_2_O)_*n*_ complexes (*n* = 10–12, 14, 15). In each case photodissociation events are due to sequential loss of either (i) loss of intact water molecules *m*H_2_O, (ii) loss of H + *x*H_2_O, and (iii) loss of H_2_ + *y*H_2_O, represented as dashed red, blue, and green lines, respectively.

For *n* = 11, bands assigned to single-acceptor water motifs around 3200 cm^−1^ increase in intensity, as presented in Fig. 7. In terms of fragmentation channels, the H_2_ + H_2_O channel (green dashed spectrum) is as intense as the H + H_2_O channel (blue dashed spectrum). However, the onset of H_2_ loss starts at lower photon energies (2890 cm^−1^) than H loss (3120 cm^−1^). Below 2890 cm^−1^, only the H_2_O fragment channel is detected. The dissociation patterns suggest that intact and inserted isomers still co-exist for this cluster size.

Going to V^+^(H_2_O)_12_, differences in fragmentation channels are observed (Fig. 7). In the whole spectral range, the H_2_ + H_2_O channel is the most intense with no H + H_2_O loss observed and water loss observed as a weaker channel. Additionally, the spectral band intensities change, indicating a completed structural change to the inserted clusters. The band at 2900 cm^−1^ for *n* = 9, 10 and 11, seems to decrease in intensity but it is still observable above the noise level. Since V^+^(H_2_O)_12_ shows the second highest BIRD rate for H_2_ + H_2_O loss, the signal-to-noise ratio, especially in the lower wavelength region, is lower due to BIRD dissociation, even at low temperatures.^19^ It is possible that this band becomes lost in the noise and thus, is undetectable. Given that H_2_ + H_2_O is the most intense channel, the majority of ions present for *n* = 12 are composed of inserted isomers. This would indicate that 12 water molecules are enough to form three dimensional structures, whereby the water network can fold back and attack the metal center, cleaving the O–H bond, forming a HVOH^+^ moiety.

The IRMPD spectra of V^+^(H_2_O)_14_ and V^+^(H_2_O)_15_ are qualitatively similar to the observed spectrum of *n* = 12 (Fig. 7). One broad absorption feature from 2900–3600 cm^−1^ along with a band within the isolated O–H stretching region around 3700 cm^−1^ is observed. The H_2_ + H_2_O loss channel decreases for *n* = 14 when compared to *n* = 12, with the fragmentation intensity decreasing sharply at *n* = 15, consistent with BIRD studies for this cluster size.^19^ It is likely that the majority of ions at cluster sizes *n* = 14, 15 are inserted, however the loss of water molecules is energetically and/or entropically preferred. Thus, although the H_2_ + H_2_O channel decreases upon increasing cluster size, these isomers are most likely still inserted, consistent with ultraviolet/visible spectroscopy of these cluster sizes.^63^

The largest clusters (up to *n* = 51) investigated are shown in Fig. 1. In this cluster size range, only water loss is observed after irradiation. For *n* = 20–51, the IRMPD spectra are qualitatively similar to those observed for *n* = 14, 15; one broad absorption feature between 2900–3600 cm^−1^ with an additional band within the free O–H stretching region around 3700 cm^−1^. These spectra, however, are less structured. The onset of the broad feature varies with the cluster size. V^+^(H_2_O)_20,25,30_ show an onset at about 2700 cm^−1^ while for clusters greater than 30 H_2_O molecules the onset is blue shifted to roughly 2900 cm^−1^. This is in stark contrast to previous experiments,^62^ where fragmentation in this size range was observed only above 3200 cm^−1^. As the *n* = 12 cluster is mostly composed of inserted isomers, this indicates that larger clusters are also composed of inserted species, in agreement with our UV/Vis spectra.^63^ This is not surprising, as this species provides the nucleation site to which additional water molecules bind, forming larger clusters. A counter example to this, revealed by UV/Vis spectroscopy, was found for hydrated magnesium cations, which also show the presence of inserted isomers for *n* = 12, (Mg^+^OH(H_2_O)_*n*−1_), however with increasing cluster size (*n* > 25) form intact structures, Mg^+^(H_2_O)_*n*_.^30^ More revealing in the large size range of V^+^(H_2_O)_*n*_ are spectra in the UV/Vis range, which do not show photodissociation, meaning the HVOH^+^(H_2_O)_*n*_ structure is present. Also, theoretical work on hydrated aluminum showed that the insertion reaction is not affected by an increasing solvation shell.^88^

The signature of a mobile proton at *ca.* 2800–3500 cm^−1^ is not visible for *n* ≥ 42.^89^ This could be due to the increased number of strong single- and double-acceptor O–H stretching bands in addition to a lower signal-to-noise ratio due to a smaller ion intensity and higher rate of BIRD. This also explains why the free O–H stretching bands around 3700 cm^−1^ decrease with increasing cluster size relative to the absorption maximum.

## Conclusions

The IRMPD spectral fingerprints and size-dependent fragmentation channels of V^+^(H_2_O)_*n*_ yield detailed insight into the cluster structure. For *n* = 3–7, structured IR spectra are observed with water loss as the only observed channel, indicating intact clusters present. H_2_ loss is observed as a weak channel at *n* = 8, indicating the onset of inserted clusters, HVOH^+^(H_2_O)_*n*−1_. The H loss channel is observed for *n* = 9–11 and arises at the point where H and H_2_O dissociation channels become energetically competitive for the intact clusters. A noticeable change in the spectroscopic pattern and fragmentation channels between *n* = 11 and 12 suggests that structural changes are responsible for preventing the release of H, *i.e.*, the clusters are fully converted to the inserted isomeric form. Finally, the H_2_ loss channel disappears around *n* = 15, likely because of the growing H_2_O network; the number of possible pathways for the mobile proton increases and the probability for it to reach the hydride site required for recombination decreases. However, since only selected cluster sizes were probed for *n* > 12, cluster sizes *n* = 17 and 18 may also show H_2_ loss upon IRMPD, as observed in previous BIRD studies. In any event, significant changes in IRMPD spectra are not observed for *n* = 14–51. Consistent with the earlier UV/Vis results, these clusters are fully transferred to their inserted isomeric form.

## Conflicts of interest

There are no conflicts of interest to declare.

## Supplementary Material

CP-024-D2CP00833E-s001

CP-024-D2CP00833E-s002

## References

[cit1] Duncan M. A. (2003). Int. Rev. Phys. Chem..

[cit2] Duncan M. A. (1997). Annu. Rev. Phys. Chem..

[cit3] Stace A. J. (2002). J. Phys. Chem. A.

[cit4] Bondybey V. E., Beyer M. K. (2002). Int. Rev. Phys. Chem..

[cit5] Beyer M. K. (2007). Mass Spectrom. Rev..

[cit6] Polfer N. C., Oomens J. (2009). Mass Spectrom. Rev..

[cit7] Artero V., Chavarot-Kerlidou M., Fontecave M. (2011). Angew. Chem., Int. Ed..

[cit8] Tard C., Pickett C. J. (2009). Chem. Rev..

[cit9] Canaguier S., Artero V., Fontecave M. (2008). Dalton Trans..

[cit10] Barton B. E., Whaley C. M., Rauchfuss T. B., Gray D. L. (2009). J. Am. Chem. Soc..

[cit11] Brimblecombe R., Swiegers G. F., Dismukes G. C., Spiccia L. (2008). Angew. Chem., Int. Ed..

[cit12] Fuke K., Hashimoto K., Iwata S. (1999). Adv. Chem. Phys..

[cit13] Niedner-Schatteburg G., Bondybey V. E. (2000). Chem. Rev..

[cit14] Donald W. A., Leib R. D., O'Brien J. T., Holm A. I. S., Williams E. R. (2008). Proc. Natl. Acad. Sci. U. S. A..

[cit15] Dalleska N. F., Honma K., Sunderlin L. S., Armentrout P. B. (1994). J. Am. Chem. Soc..

[cit16] Balaj O. P., Berg C. B., Reitmeier S. J., Bondybey V. E., Beyer M. K. (2009). Int. J. Mass Spectrom..

[cit17] Beyer M., Berg C., Görlitzer H. W., Schindler T., Achatz U., Albert G., Niedner-Schatteburg G., Bondybey V. E. (1996). J. Am. Chem. Soc..

[cit18] Beyer M., Achatz U., Berg C., Joos S., Niedner-Schatteburg G., Bondybey V. E. (1999). J. Phys. Chem. A.

[cit19] Fox B. S., Balteanu I., Balaj O. P., Liu H. C., Beyer M. K., Bondybey V. E. (2002). Phys. Chem. Chem. Phys..

[cit20] van der Linde C., Beyer M. K. (2012). J. Phys. Chem. A.

[cit21] van der Linde C., Beyer M. K. (2011). Phys. Chem. Chem. Phys..

[cit22] Scharfschwerdt B., van der Linde C., Balaj O. P., Herber I., Schütze D., Beyer M. K. (2012). Low Temp. Phys..

[cit23] Lessen D. E., Asher R. L., Brucat P. J. (1990). J. Chem. Phys..

[cit24] Rosi M., Bauschlicher C. W. (1989). J. Chem. Phys..

[cit25] Rosi M., Bauschlicher C. W. (1990). J. Chem. Phys..

[cit26] Farrar J. M. (2003). Int. Rev. Phys. Chem..

[cit27] Yeh C. S., Willey K. F., Robbins D. L., Pilgrim J. S., Duncan M. A. (1992). Chem. Phys. Lett..

[cit28] Scurlock C. T., Pullins S. H., Reddic J. E., Duncan M. A. (1996). J. Chem. Phys..

[cit29] Ončák M., Taxer T., Barwa E., van der Linde C., Beyer M. K. (2018). J. Chem. Phys..

[cit30] Taxer T., Ončák M., Barwa E., van der Linde C., Beyer M. K. (2019). Faraday Discuss..

[cit31] Daluz J. S., Kocak A., Metz R. B. (2012). J. Phys. Chem. A.

[cit32] Kocak A., Austein-Miller G., Pearson W. L., Altinay G., Metz R. B. (2013). J. Phys. Chem. A.

[cit33] Abate Y., Kleiber P. D. (2005). J. Chem. Phys..

[cit34] Sanekata M., Misaizu F., Fuke K. (1996). J. Chem. Phys..

[cit35] Iino T., Ohashi K., Inoue K., Judai K., Nishi N., Sekiya H. (2007). Eur. Phys. J. D.

[cit36] Bandyopadhyay B., Reishus K. N., Duncan M. A. (2013). J. Phys. Chem. A.

[cit37] Lisy J. M. (1997). Int. Rev. Phys. Chem..

[cit38] Beck J. P., Lisy J. M. (2011). J. Chem. Phys..

[cit39] Walker N. R., Walters R. S., Tsai M.-K., Jordan K. D., Duncan M. A. (2005). J. Phys. Chem. A.

[cit40] Walters R. S., Brinkmann N. R., Schaefer H. F., Duncan M. A. (2003). J. Phys. Chem. A.

[cit41] Walters R. S., Pillai E. D., Duncan M. A. (2005). J. Am. Chem. Soc..

[cit42] Vaden T. D., Lisy J. M., Carnegie P. D., Pillai E. D., Duncan M. A. (2006). Phys. Chem. Chem. Phys..

[cit43] Carnegie P. D., McCoy A. B., Duncan M. A. (2009). J. Phys. Chem. A.

[cit44] Carnegie P. D., Bandyopadhyay B., Duncan M. A. (2011). J. Chem. Phys..

[cit45] Carnegie P. D., Bandyopadhyay B., Duncan M. A. (2008). J. Phys. Chem. A.

[cit46] Carnegie P. D., Bandyopadhyay B., Duncan M. A. (2011). J. Phys. Chem. A.

[cit47] Bandyopadhyay B., Duncan M. A. (2012). Chem. Phys. Lett..

[cit48] Iino T., Ohashi K., Mune Y., Inokuchi Y., Judai K., Nishi N., Sekiya H. (2006). Chem. Phys. Lett..

[cit49] Inokuchi Y., Ohshimo K., Misaizu F., Nishi N. (2004). J. Phys. Chem. A.

[cit50] Inokuchi Y., Ohshimo K., Misaizu F., Nishi N. (2004). Chem. Phys. Lett..

[cit51] Iino T., Ohashi K., Inoue K., Judai K., Nishi N., Sekiya H. (2007). J. Chem. Phys..

[cit52] Furukawa K., Ohashi K., Koga N., Imamura T., Judai K., Nishi N., Sekiya H. (2011). Chem. Phys. Lett..

[cit53] Bush M. F., Saykally R. J., Williams E. R. (2008). J. Am. Chem. Soc..

[cit54] O'Brien J. T., Williams E. R. (2008). J. Phys. Chem. A.

[cit55] Cunningham E. M., Taxer T., Heller J., Ončák M., van der Linde C., Beyer M. K. (2021). Phys. Chem. Chem. Phys..

[cit56] Asmis K. R. (2012). Phys. Chem. Chem. Phys..

[cit57] Schwarz H., Asmis K. R. (2019). Chem. – Eur. J..

[cit58] Sasaki J., Ohashi K., Inoue K., Imamura T., Judai K., Nishi N., Sekiya H. (2009). Chem. Phys. Lett..

[cit59] Zhou M., Dong J., Zhang L., Qin Q. (2001). J. Am. Chem. Soc..

[cit60] Walker N. R., Walters R. S., Pillai E. D., Duncan M. A. (2003). J. Chem. Phys..

[cit61] Ward T. B., Miliordos E., Carnegie P. D., Xantheas S. S., Duncan M. A. (2017). J. Chem. Phys..

[cit62] Carnegie P. D., Marks J. H., Brathwaite A. D., Ward T. B., Duncan M. A. (2020). J. Phys. Chem. A.

[cit63] Heller J., Pascher T. F., Muß D., van der Linde C., Beyer M. K., Ončák M. (2021). Phys. Chem. Chem. Phys..

[cit64] Heller J., Tang W. K., Cunningham E. M., Demissie E. G., van der Linde C., Lam W. K., Ončák M., Siu C.-K., Beyer M. K. (2021). Angew. Chem., Int. Ed..

[cit65] Akhgarnusch A., Tang W. K., Zhang H., Siu C.-K., Beyer M. K. (2016). Phys. Chem. Chem. Phys..

[cit66] Allemann M., Kellerhals H., Wanczek K. P. (1983). Int. J. Mass Spectrom. Ion Process..

[cit67] Berg C., Schindler T., Niedner-Schatteburg G., Bondybey V. E. (1995). J. Chem. Phys..

[cit68] Akhgarnusch A., Höckendorf R. F., Beyer M. K. (2015). J. Phys. Chem. A.

[cit69] Caravatti P., Allemann M. (1991). Org. Mass Spectrom..

[cit70] Bondybey V. E., English J. H. (1981). J. Chem. Phys..

[cit71] Dietz T. G., Duncan M. A., Powers D. E., Smalley R. E. (1981). J. Chem. Phys..

[cit72] Proch D., Trickl T. (1989). Rev. Sci. Instrum..

[cit73] Marshall A. G., Hendrickson C. L., Jackson G. S. (1998). Mass Spectrom. Rev..

[cit74] Polfer N., Sartakov B. G., Oomens J. (2004). Chem. Phys. Lett..

[cit75] Heller J., Cunningham E. M., van der Linde C., Ončák M., Beyer M. K. (2022). J. Phys. Chem. Lett..

[cit76] Wong R. L., Paech K., Williams E. R. (2004). Int. J. Mass Spectrom..

[cit77] Thölmann D., Tonner D. S., McMahon T. B. (1994). J. Phys. Chem..

[cit78] Dunbar R. C. (2004). Mass Spectrom. Rev..

[cit79] Schindler T., Berg C., Niedner-Schatteburg G., Bondybey V. E. (1996). Chem. Phys. Lett..

[cit80] Schnier P. D., Price W. D., Jockusch R. A., Williams E. R. (1996). J. Am. Chem. Soc..

[cit81] Sena M., Riveros J. M. (1994). Rapid Commun. Mass Spectrom..

[cit82] Fox B. S., Beyer M. K., Bondybey V. E. (2001). J. Phys. Chem. A.

[cit83] Frisch M. J. , Trucks G. W., Schlegel H. B., Scuseria G. E., Robb M. A., Cheeseman J. R., Scalmani G., Barone V., Petersson G. A., Nakatsuji H., Li X., Caricato M., Marenich A. V., Bloino J., Janesko B. G., Gomperts R., Mennucci B., Hratchian H. P., Ortiz J. V., Izmaylov A. F., Sonnenberg J. L., Williams-Young D., Ding F., Lipparini F., Egidi F., Goings J., Peng B., Petrone A., Henderson T., Ranasinghe D., Zakrzewski V. G., Gao J., Rega N., Zheng G., Liang W., Hada M., Ehara M., Toyota K., Fukuda R., Hasegawa J., Ishida M., Nakajima T., Honda Y., Kitao O., Nakai H., Vreven T., Throssell K., Montgomery, Jr. J. A., Peralta J. E., Ogliaro F., Bearpark M. J., Heyd J. J., Brothers E. N., Kudin K. N., Staroverov V. N., Keith T. A., Kobayashi R., Normand J., Raghavachari K., Rendell A. P., Burant J. C., Iyengar S. S., Tomasi J., Cossi M., Millam J. M., Klene M., Adamo C., Cammi R., Ochterski J. W., Martin R. L., Morokuma K., Farkas O., Foresman J. B. and Fox D. J., Gaussian 16 Revision A.03, 2016

[cit84] Cunningham E. M., Taxer T., Heller J., Ončák M., van der Linde C., Beyer M. K. (2021). Int. J. Mol. Sci..

[cit85] Kim J., Lee S., Cho S. J., Mhin B. J., Kim K. S. (1995). J. Chem. Phys..

[cit86] Ohashi K., Sasaki J., Yamamoto G., Judai K., Nishi N., Sekiya H. (2014). J. Chem. Phys..

[cit87] Reinhard B. M., Niedner-Schatteburg G. (2002). J. Phys. Chem. A.

[cit88] Siu C.-K., Liu Z.-F., Tse J. S. (2002). J. Am. Chem. Soc..

[cit89] Johnson C. J., Dzugan L. C., Wolk A. B., Leavitt C. M., Fournier J. A., McCoy A. B., Johnson M. A. (2014). J. Phys. Chem. A.

