# Supplementary material for: Size-dependent H and H2 formation by infrared multiple photon dissociation spectroscopy of hydrated vanadium cations, V+(H2O)n, n = 3–51
Source: Phys Chem Chem Phys. 2022 Apr 6;24(24):14699–708. doi: 10.1039/d2cp00833e (PMC9215701; doi:10.1039/d2cp00833e)
Supplement: CP-024-D2CP00833E-s002 [file CP-024-D2CP00833E-s002.pdf]

Electronic Supplementary Material (ESI) for Physical Chemistry Chemical Physics.
This journal is © the Owner Societies 2022

Supporting Information

Size-Dependent H and H_2 Formation by Infrared Multiple Photon Dissociation Spectroscopy of Hydrated Vanadium Cations, $V^+(H_2O)_n$, n = 3–51

Jakob Heller, Ethan M. Cunningham, Jessica C. Hartmann, Christian van der Linde, Milan Ončák,* and Martin K. Beyer*

Institut für Ionenphysik und Angewandte Physik, Universität Innsbruck, Technikerstraße 25, 6020 Innsbruck, Austria

Corresponding authors: milan.oncak@uibk.ac.at, martin.beyer@uibk.ac.at

Contents

IRMPD Spectra of Small Clusters 2
Ligand Fragmentation 3
References 4
Cartesian structures of optimized ions (Å) along with ZPE-corrected energies (Hartree) 4
IR frequencies (in cm^{-1}, without shift) and intensities (KM/mole) for optimized structures 12

IRMPD Spectra of Small Clusters

The measured IRMPD spectra below, along with the simulated structures and spectra, are consistent with findings from Ohashi and Duncan.[1,2] A direct comparison between IRMPD spectra of the present study with those measured by Duncan can be found in Figure 1 of the main article.

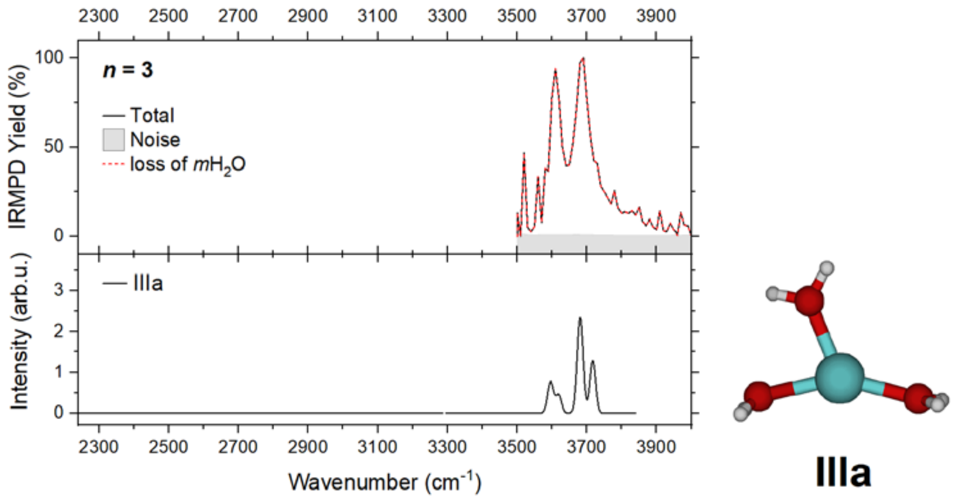

Figure S1. Infrared multiple photon dissociation spectrum of the $V^+(H_2O)_3$ complex along with the simulated spectra of the low-lying intact (**IIIa**) isomer calculated at the B3LYP/aug-cc-pVDZ level of theory.

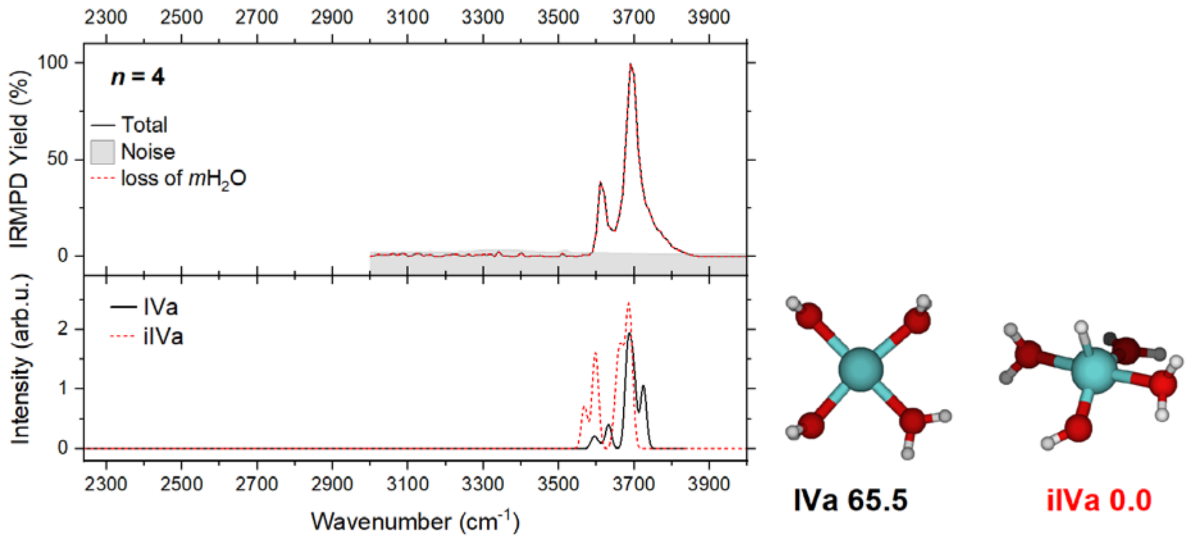

Figure S2. Infrared multiple photon dissociation spectrum of the $V^+(H_2O)_4$ complex along with the simulated spectra of the low-lying intact (**IVa**) and inserted (**iIVa**) isomers calculated at the B3LYP/aug-cc-pVDZ level of theory. Structural isomers also show relative energies given in kJ mol^{-1} inclusive of zero-point energy.

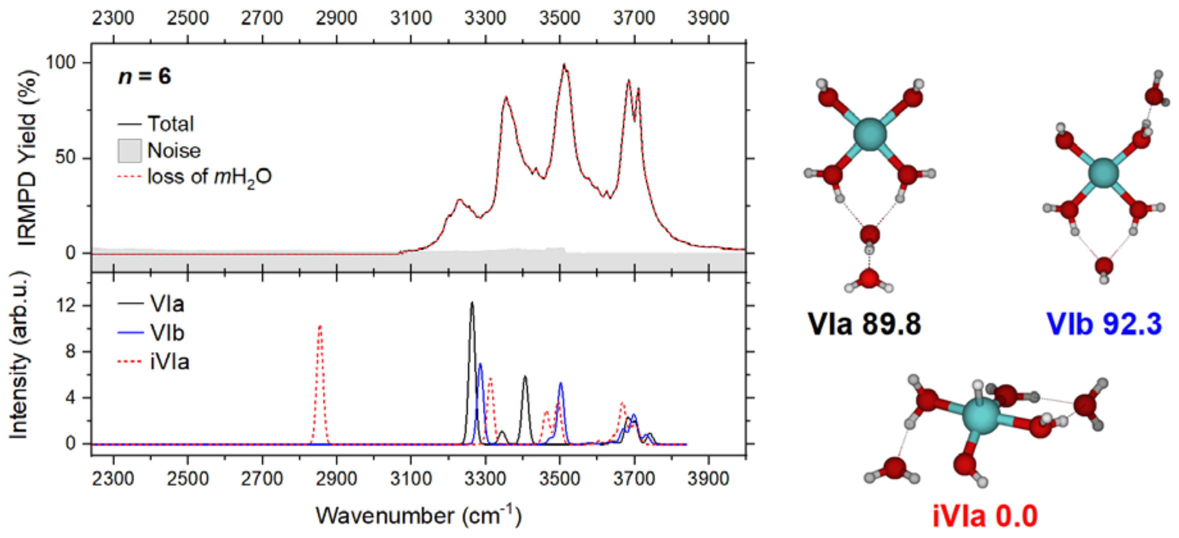

Figure S3. Infrared multiple photon dissociation spectrum of the $V^+(H_2O)_6$ complex along with the simulated spectra of the low-lying intact (**VIa, VIb**) and inserted (**iVIa**) isomers calculated at the B3LYP/aug-cc-pVDZ level of theory. Structural isomers also show relative energies given in kJ mol^{-1} inclusive of zero-point energy.

Ligand Fragmentation

Table S1 – Number of ligands lost during the IRMPD process for each cluster size, $V^+(H_2O)_n$ (n = 3–12, 14, 15, 20, 25, 30, 42 and 51), consistent with measured black body infrared dissociation (BIRD) values.[3]

$V^+(H_2O)_n$	$m(H_2O)$	H + $x(H_2O)$	H_2 + $y(H_2O)$
3	1	–	–
4	1	–	–
5	1	–	–
6	1,2	–	–
7	1,2	–	–
8	1,2	–	1
9	1,2	0,1	1,2
10	1,2	0,1	2
11	1,2	0,1	1,2
12	1	–	2,3
14	1	–	2,3,4
15	1,2,3	–	3
20	1,2,3,4	–	–
25	1,2,3,4	–	–
30	1,2,3,4,5	–	–
42	1,2,3	–	–
51	1,2,3	–	–

References

1 J. Sasaki, K. Ohashi, K. Inoue, T. Imamura, K. Judai, N. Nishi and H. Sekiya, *Chem. Phys. Lett.*, 2009, **474**, 36.
2 P. D. Carnegie, J. H. Marks, A. D. Brathwaite, T. B. Ward and M. A. Duncan, *J. Phys. Chem. A*, 2020, **124**, 1093.
3 B. S. Fox, I. Balteanu, O. P. Balaj, H. C. Liu, M. K. Beyer and V. E. Bondybey, *Phys. Chem. Chem. Phys.*, 2002, **4**, 2224.

Cartesian structures of optimized ions (Å) along with ZPE-corrected energies (Hartree)

```
IIIa
E = -1173.103678
O 2.330794 -0.132725 -0.000142
V 0.213123 -0.142249 0.000179
O -1.058361 1.591990 -0.000542
O -1.739995 -1.130670 0.000002
H -2.062824 -1.611282 -0.775974
H -2.061265 -1.615427 0.774030
H 2.903368 -0.096855 -0.780030
H 2.903689 -0.096167 0.779475
H -2.023711 1.535106 0.002890
H -0.820593 2.527587 0.000955

IVa
E = -1249.557986
O -0.521866 -2.071034 0.000138
V 0.004612 0.029990 -0.000030
O -2.159259 0.417555 -0.000289
O 0.569618 2.148692 0.000194
O 2.091808 -0.657801 0.000130
H 0.098495 -2.810769 0.002927
H -1.414797 -2.437914 -0.001204
H 2.662546 -0.544535 0.773527
H -2.625454 0.759361 0.775737
H 2.659965 -0.551879 -0.776211
H -2.624621 0.762328 -0.775499
H 0.645717 2.717683 0.777783
H 0.649667 2.716649 -0.777759

iIVa
E = -1249.582933
O -2.114704 -0.203523 0.193675
V -0.012727 0.103277 0.211674
O 0.246513 -1.841736 -0.462338
O -0.356143 1.637637 -0.658842
O 2.131221 0.199309 0.196030
H -2.586129 -0.315844 1.032578
H -2.519960 0.561016 -0.249296
H 0.257915 2.338005 -0.917522
H -0.462381 -2.439138 -0.742144
H 2.511194 0.401631 1.064365
H 2.654864 0.685668 -0.457349
H 0.086844 0.347917 1.848531
H 1.095281 -2.288119 -0.595873

Va
E = -1326.000340
O 2.041927 -1.655097 0.014051
V 0.597029 0.000103 0.000988
O -0.986991 -1.473599 -0.049081
O 2.042016 1.655169 0.013784
O -0.987281 1.473579 -0.047125
H 2.547058 -1.917840 -0.768255
H 2.578689 -1.883183 0.785862
H -1.928787 -1.230014 0.007023
H 2.554802 1.911434 -0.765685
H -0.904265 -2.427076 0.066670
H 2.572904 1.886046 0.788860
H -1.929803 1.232008 0.004805
H -0.904174 2.428879 0.051789
O -3.402052 -0.000225 0.035025
H -3.991535 -0.001638 -0.732394
H -3.987505 0.000406 0.805363

Vb
E = -1325.997426
O 3.679063 -0.141104 -0.608385
V -0.602970 0.027119 0.137021
O 1.426203 0.511915 0.715977
O -0.891302 2.181174 -0.122546
O -0.269479 -2.134473 0.227668
O -2.631020 -0.471648 -0.567220
H 4.341544 0.490471 -0.915156
H 3.944892 -1.000684 -0.957409
H -0.090278 2.724024 -0.116070
H 0.093274 -2.575612 1.007813
H -1.567806 2.689253 0.345861
H -0.937443 -2.730489 -0.138327
H -2.951829 -0.170668 -1.429393
H -3.393903 -0.469867 0.028664
H 2.236982 0.248782 0.216892
H 1.685165 0.604141 1.641692

Vc
E = -1325.988819
O 1.903491 -0.021790 -0.266750
H 2.602523 0.590917 -0.525972
H 2.298538 -0.921798 -0.179162
V -0.051691 0.662188 0.202049
O 0.902576 2.570157 -0.362874
H 0.629493 3.025146 -1.172329
H 1.029008 3.257453 0.306776
O -1.815029 -0.232689 0.844801
H -2.550135 -0.612185 0.296120
H -2.047778 -0.359470 1.773698
O 2.929349 -2.488822 -0.018927
H 3.456766 -2.797823 0.728331
H 3.138644 -3.075273 -0.756692
O -3.779523 -1.247184 -0.621195
H -3.893773 -2.175299 -0.860498
```

H -4.601304 -0.799358 -0.857824

iVa
E = -1326.030297
O 0.495591 -1.884174 0.384740
V -0.670272 -0.115809 0.231817
O -1.765206 1.733748 0.074854
O 0.975251 1.044336 -0.032199
O -1.665100 -1.088987 -0.921599
O 3.476941 0.348447 -0.265727
H 0.667418 -2.273675 1.253937
H 0.023312 -2.556215 -0.134890
H 1.934231 0.768371 -0.128639
H 0.917138 2.004262 -0.128176
H -2.490577 -0.845600 -1.360088
H -2.291558 1.914016 0.868037
H 3.911121 0.087698 -1.088053
H 4.178048 0.559045 0.364239
H -1.257398 -0.194418 1.785997
H -2.315294 1.973173 -0.684717

iVb
E = -1326.028389
O 1.189867 1.603017 -0.763277
V 0.443702 -0.006115 0.307703
O -0.487166 -1.493618 -0.178824
O -1.248969 1.150815 0.632433
O 2.388194 -0.867001 -0.097285
O -3.004182 -0.308127 -0.527930
H 2.079577 1.712736 -1.127097
H 0.667656 2.394560 -0.959606
H -2.077196 0.696613 0.268856
H 2.889112 -1.071783 0.706144
H -1.437299 1.427347 1.539800
H 2.571792 -1.574655 -0.731575
H -2.469459 -1.121950 -0.513840
H -0.110442 -2.375930 -0.296053
H -3.926540 -0.550295 -0.381165
H 0.905685 -0.076679 1.896433

iVc
E = -1326.022934
O 1.876631 -1.303453 -0.595273
V 0.386890 -0.071533 0.248903
O -1.010334 1.414799 0.157512
O -0.774039 -1.456155 0.461519
O 1.880314 1.439375 -0.260981
O -2.983137 -0.006926 -0.623936
H 1.711810 -2.252090 -0.690784
H 2.831670 -1.162536 -0.539247
H -2.698193 -0.872696 -0.286471
H 1.831504 2.092652 -0.973271
H -0.796741 -1.972544 1.279553
H 2.335907 1.855805 0.484219
H -1.912100 1.037415 -0.099422
H -1.124537 2.044254 0.882046
H -3.946979 0.004830 -0.661367
H 0.953696 0.169055 1.769253

VIa
E = -1402.439937
O 2.664644 -1.651958 -0.258946
V 1.235792 0.000150 -0.002736
O -0.339140 1.458748 0.223022
O -0.339372 -1.458199 0.223124
O 2.665848 1.651059 -0.259498
O -2.588223 0.000488 0.854852
H 3.001374 -1.922740 -1.124699
H 3.351458 -1.860521 0.389448
H -1.236606 -1.169584 0.493389
H 3.002816 1.920943 -1.125439
H -0.238916 -2.396321 0.419086
H 3.353125 1.858752 0.388684
H -1.236481 1.170466 0.493291
H -0.238385 2.396859 0.418905
H -3.370192 0.000445 0.252607
H -2.950341 0.000542 1.750209
O -4.742317 -0.000295 -0.788532
H -5.216485 0.771013 -1.122606
H -5.216105 -0.772039 -1.122135

VIb
E = -1402.438963
O 1.804480 -0.724175 0.796868
V -0.000826 0.321270 0.199891
O -1.880556 1.188439 -0.463432
O -1.042011 -1.581518 0.182914
O 0.865104 2.340090 0.074250
O -3.766093 -0.932472 -0.275448
H 2.618916 -0.814780 0.249593
H 2.072083 -0.777553 1.722821
H -2.011268 -1.641636 0.109322
H 1.480678 2.561099 -0.639677
H -0.703777 -2.413927 0.532717
H 1.194120 2.780631 0.870804
H -2.710763 0.680430 -0.422650
H -2.095171 2.128285 -0.431427
H -4.193759 -1.240542 -1.086882
H -4.449678 -0.982713 0.407222
O 4.002839 -0.948596 -0.796457
H 4.023252 -1.591884 -1.516507
H 4.924263 -0.810760 -0.542390

iVIa
E = -1402.474132
O 1.150201 -1.347323 0.488387
V 0.129463 -0.204151 -0.500786
O -1.042682 1.366279 0.113934
O -1.638537 -1.411632 -0.255729
O 1.684835 1.175433 -0.707961
O 3.347536 0.223112 1.006091
O -3.549164 0.506645 0.720614
H 0.128985 -0.643856 -2.102375
H 0.784333 -2.181201 0.814872
H 2.021064 1.323629 -1.602001
H 2.471794 0.931750 -0.122941
H -0.692187 2.251857 0.280104
H -1.981220 1.300467 0.401056
H -2.482014 -1.010708 0.023052
H -1.826880 -2.012109 -0.989968
H -3.859118 0.432145 1.635110
H -4.321182 0.797328 0.213396
H 2.867887 -0.627547 1.039823
H 4.293376 0.033609 1.005253

VIIIa
E = -1555.316971
O 1.508037 1.533991 -0.062419
V -0.044503 -0.002970 -0.214097
O -1.596065 1.521618 -0.405458
O 1.499213 -1.546646 -0.046243
O -1.600953 -1.524083 -0.386914
O -3.908361 0.000365 -0.630597
O -5.696331 0.013338 1.409869
O 3.784584 -0.016500 -0.494393
H 1.512925 2.113755 0.711044
H 2.418747 1.184327 -0.165198
H -2.507374 1.184120 -0.538464
H 1.501076 -2.112473 0.737547
H -1.469719 2.259854 -1.015156
H 2.411437 -1.203022 -0.154393
H -2.511012 -1.185324 -0.524808
H -1.476462 -2.271027 -0.986291
H -4.546132 0.006419 0.122093
H -4.452068 -0.001658 -1.428664
H -6.016711 -0.755231 1.897639
H -6.019860 0.787947 1.885859
H 4.605072 -0.003152 0.051588
H 4.086267 -0.008393 -1.412466
O 6.053250 0.020321 1.014646

H 6.555073 0.797351 1.289621
H 6.585321 -0.744421 1.266344

VIIIb
E = -1555.316095
O -1.110662 -1.810721 -0.511910
V 0.535772 -0.439403 -0.077980
O 1.936772 -2.079527 0.317692
O -0.802394 1.195185 -0.544840
O 2.090560 0.989704 0.449744
O -3.270618 -0.069803 -0.812652
O 4.430331 -0.680286 0.133269
O 0.807824 3.525193 0.054661
H -1.964133 -1.361347 -0.692225
H -0.990466 -2.480180 -1.198082
H 2.872145 -1.801689 0.348776
H -1.766524 1.048693 -0.577676
H 1.778780 -2.622877 1.102255
H -0.602420 2.112374 -0.302277
H 3.020204 0.762939 0.284572
H 1.945634 1.932693 0.261107
H 5.139987 -0.707149 0.790064
H 4.870207 -0.799782 -0.720130
H -3.979041 -0.121268 -0.130952
H -3.731433 0.016092 -1.656876
H 0.739229 4.082603 0.841888
H 0.971762 4.137815 -0.675349
O -5.243834 -0.253645 1.064214
H -5.565024 -1.075388 1.455134
H -5.765496 0.453959 1.461890

VIIIc
E = -1555.315237
O 0.686858 -2.062732 -0.285193
V 0.000002 -0.000154 -0.200514
O 2.062764 0.686432 -0.284834
O -2.062674 -0.686645 -0.285331
O -0.686671 2.062523 -0.286079
O -1.745661 -3.487771 0.322506
O 1.745246 3.487971 0.322504
O -3.488128 1.745375 0.322002
H 0.081266 -2.754785 0.029918
H 1.585649 -2.253926 0.031987
H 2.253771 1.585267 0.032395
H -2.253835 -1.585399 0.032043
H 2.754025 0.080534 0.031600
H -2.754401 -0.080860 0.030235
H -0.080872 2.754561 0.028784
H -1.585359 2.253850 0.031387
H 1.959281 3.919638 1.160797
H 2.052001 4.098064 -0.362777
H -1.961007 -3.919094 1.160642
H -2.051629 -4.098019 -0.362994
H -3.919913 1.960805 1.159874
H -4.097936 2.051402 -0.363857
O 3.488314 -1.744861 0.321627
H 4.097264 -2.051159 -0.364881
H 3.921271 -1.959669 1.159059

VIIId
E = -1555.314445
O -2.967198 1.265891 -0.727024
V -1.306495 0.042537 0.024518
O -0.019650 1.792819 0.115931
O -2.565881 -1.754170 -0.052397
O 0.372006 -1.123098 0.767067
O 2.327359 0.770911 1.095911
O 4.451761 0.726594 -0.467366
O -5.149854 -0.507767 -0.156067
H -2.897674 1.643337 -1.615015
H -3.847947 0.851505 -0.661875
H 0.879364 1.638296 0.484686
H -2.357358 -2.434913 -0.707356
H -0.339028 2.633784 0.465594
H -3.524148 -1.582530 -0.115841
H 1.184068 -0.612028 0.984180
H 0.258442 -1.796128 1.449307
H 3.126781 0.747100 0.500624
H 2.661614 1.019023 1.966782
H 5.139020 0.030534 -0.528233
H 4.710928 1.430203 -1.071811
H -5.889721 -0.735917 -0.736305
H -5.546540 -0.364238 0.715145
O 6.395140 -1.220020 -0.604683
H 7.253417 -1.171729 -0.166177
H 6.488711 -1.883947 -1.298597

iVIIIa
E = -1555.359085
V 0.173830 -0.584076 0.273901
H 1.062098 -1.644020 1.222947
O -0.506090 -1.294835 -1.283632
H 0.039110 -1.261685 -2.081847
O -1.574331 -1.191907 1.315806
H -1.482141 -1.978341 1.868701
H -2.354712 -1.313751 0.727245
O -0.304412 1.328959 0.707370
H -1.277860 1.567428 0.615981
H 0.249258 2.065796 0.378251
O 1.950050 0.107469 -0.602102
H 2.148761 1.059419 -0.612714
H 2.777167 -0.388051 -0.363245
O 4.068179 -1.272350 0.206029
H 3.826533 -1.940147 0.861612
H 4.786629 -1.651582 -0.315766
O 1.707000 2.966714 -0.325958
H 1.592466 3.534492 -1.100839
H 2.247805 3.481827 0.289150
O -3.131296 -0.929230 -0.846899
H -2.287086 -1.152179 -1.310427
H -3.847190 -1.389183 -1.301635
O -2.809769 1.717376 0.133024
H -3.152718 0.897261 -0.275006
H -3.520854 2.088879 0.668768

iVIIIb
E = -1555.357661
O -1.244175 -0.331732 1.410176
V 0.586194 -0.824439 0.522323
O 2.508694 -1.137688 -0.402562
O 0.989604 1.165394 0.411362
O -0.258860 -1.888732 -0.723541
O -1.314989 2.375316 0.720005
O -2.795790 1.411890 -1.251020
O -2.853735 -1.302094 -0.532571
O 3.477083 1.499627 -0.814328
H -2.014066 -0.738769 0.947787
H -1.446482 0.620040 1.496654
H 1.779092 1.550320 -0.015088
H -2.000577 -1.717810 -0.814552
H 0.241024 1.819361 0.484519
H 0.255603 -2.383152 -1.375503
H 3.070565 -0.364528 -0.602859
H 3.053526 -1.779916 0.072620
H 3.594127 1.821579 -1.719556
H 4.216161 1.874527 -0.314205
H -2.994926 0.459335 -1.146919
H -3.536123 -1.983713 -0.562998
H -3.452408 1.779025 -1.853275
H -1.575809 3.212875 1.121329
H -1.933047 2.190305 -0.037431
H 1.198245 -1.733229 1.765886

iVIIIc
E = -1555.357067
O 0.992103 0.134340 1.456383
V -0.710599 0.057110 0.235251
O -2.365838 -0.317608 -1.040191
O -0.110282 -1.813249 -0.297443
O -0.292881 1.698718 -0.502022

O 2.393380 -2.030880 0.360131
O 3.517724 -0.057322 -0.995227
O -4.642585 0.076107 0.224736
O 2.255173 2.160916 0.198406
H 1.555814 0.924833 1.284184
H 1.587853 -0.638905 1.445911
H -0.576150 -2.403460 -0.902668
H 1.356040 2.271556 -0.197339
H 0.834148 -2.108851 -0.146362
H -0.894128 2.185845 -1.079752
H -2.441178 0.045450 -1.931725
H -3.256908 -0.215493 -0.602222
H 3.261580 0.832341 -0.680535
H 2.565887 3.033830 0.468277
H -5.484849 -0.391470 0.161009
H -4.513810 0.286643 1.159197
H 4.317272 0.042014 -1.523962
H 2.940689 -2.775214 0.638128
H 2.956616 -1.416032 -0.182548
H -1.839452 0.205206 1.441455

iVIIId
E = -1555.353271
O 1.000912 -1.881451 -0.564151
V -0.674119 -0.703756 0.020327
O -2.200479 0.619445 0.640252
O 0.499803 0.932375 -0.334312
O -0.784227 -1.790373 1.459235
O 2.949525 0.066852 -1.017496
O 5.138373 0.374359 0.532248
O -4.261870 -0.165409 -0.811954
O -0.890277 3.232517 0.268216
H 1.109399 -2.643773 0.022189
H 1.869038 -1.448621 -0.686857
H 0.229044 1.859457 -0.176197
H 1.451789 0.847931 -0.582386
H -1.459530 -1.649162 2.137354
H -2.056047 1.577750 0.587342
H -3.069080 0.412243 0.202099
H -0.715724 3.788335 1.040407
H -1.191372 3.840782 -0.421185
H 5.702229 -0.349687 0.832405
H -5.139078 -0.497508 -0.583183
H -3.806264 -0.881694 -1.281013
H 5.653612 1.181310 0.655528
H 3.240352 0.129639 -1.936750
H 3.762883 0.189301 -0.469122
H -1.690606 -1.276430 -1.184473

IXa
E = -1631.753600
O -1.651980 -1.788061 -0.658039
V -0.045427 -0.343544 -0.299676
O 1.483359 1.127156 0.166035
O 1.506016 -1.887951 -0.227795
O -1.520382 1.231601 -0.480147
O -3.932176 -0.173734 -0.514229
O -5.630762 -0.665728 1.570943
O 3.803468 -0.314434 -0.474304
O 0.017628 3.622818 0.023190
H -2.543603 -1.379974 -0.686186
H -1.578282 -2.362040 -1.432041
H 2.410902 -1.510562 -0.266054
H -2.470436 1.032132 -0.388406
H 1.487520 -2.499457 0.520827
H -1.331385 2.143073 -0.208030
H 2.406443 0.920397 -0.072389
H 1.304062 2.070304 0.024046
H 4.591065 -0.386784 0.110345
H 4.151078 -0.293437 -1.375104
H -4.532694 -0.334248 0.248609
H -4.512457 -0.054124 -1.276709
H 0.016702 4.153234 0.831541
H 0.046090 4.264073 -0.699893
H -5.805237 -1.537687 1.945765
H -6.074755 -0.032735 2.148057
O 5.988555 -0.542422 1.162668
H 6.455911 0.173401 1.610140
H 6.514081 -1.338000 1.311463

IXb
E = -1631.753268
O -1.560878 -2.113574 -0.110562
V 0.318923 -1.001096 -0.001629
O 2.099477 0.234454 0.175623
O 1.450003 -2.738455 0.718882
O -0.727376 0.713658 -0.781237
O -3.396205 -0.149940 -0.855503
O -5.425003 0.344155 0.914595
O 4.086327 -1.904631 -0.006903
O 1.247095 2.706350 -0.830512
H -2.319794 -1.570678 -0.415339
H -1.580842 -2.939128 -0.611861
H 2.414767 -2.629730 0.611711
H -1.698339 0.765711 -0.815559
H 1.298205 -3.010205 1.635022
H -0.318169 1.596836 -0.802684
H 2.960170 -0.163324 -0.028078
H 2.049384 1.128412 -0.217446
H 4.882448 -1.961529 0.539405
H 4.342146 -2.267166 -0.866662
H -4.112255 0.047671 -0.210690
H -3.829342 -0.178972 -1.717964
H 1.327511 3.493537 -0.247388
H 1.499044 3.013682 -1.710403
H -5.839621 -0.322299 1.475992
H -5.798874 1.191460 1.184936
O 1.511263 4.921231 0.769025
H 0.876086 5.635122 0.902269
H 2.234626 5.089813 1.384953

IXc
E = -1631.752627
V -0.707014 0.232951 -0.290494
O 0.974019 -1.033394 -0.883393
O 0.721328 1.832809 0.132003
O -2.388316 1.496262 0.325778
O -2.137720 -1.356239 -0.767339
H -2.017585 -1.835594 -1.597417
H -3.077972 -1.080701 -0.730163
H -3.271174 1.099959 0.163985
H -2.387600 1.807754 1.240860
H 0.518084 2.726253 -0.172971
H 1.640982 1.633932 -0.153337
H 1.837912 -0.562010 -0.895427
H 1.111734 -1.847407 -0.381001
O 3.081823 0.728306 -0.762246
H 3.834905 0.581865 -0.127045
H 3.486020 1.020067 -1.588749
O -4.577951 -0.058291 -0.402376
H -5.237587 -0.404330 0.242753
H -5.090459 0.237877 -1.165502
O 5.036551 0.304980 0.971549
H 5.816814 -0.274577 0.844041
H 5.258837 0.915686 1.682787
O -6.392366 -1.007092 1.391364
H -6.626218 -1.933360 1.528003
H -7.011980 -0.492843 1.923216
O 7.192083 -1.365422 0.572358
H 8.029089 -1.125770 0.156119
H 7.371895 -2.160011 1.089607

IXd
E = -1631.749201
O 0.880178 1.863894 -0.137372
V 2.079762 0.043329 -0.094865
O 3.277371 -1.792734 -0.080481
O 3.912919 1.241259 0.005085
O 0.248530 -1.133279 -0.224819
O -1.572892 0.835078 -0.775665

O -3.638909 1.126521 0.807718
O -5.597252 -0.707322 0.919434
O 5.910833 -0.772285 0.385160
H 4.041601 1.951001 -0.638407
H 4.756227 0.756829 0.073785
H 4.243316 -1.690817 0.004097
H 3.118023 -2.458109 -0.763308
H 0.012271 -1.720698 0.505029
H -0.546046 -0.583585 -0.418938
H -0.067801 1.693700 -0.345677
H 0.900099 2.458021 0.624277
H -2.382968 0.960767 -0.200258
H -1.878043 0.925456 -1.686925
H 6.224217 -0.863397 1.296240
H 6.690945 -0.919387 -0.167560
H -4.007113 1.981603 1.054834
H -4.381852 0.475308 0.850486
H -5.823113 -1.152614 1.743537
H -6.408848 -0.713256 0.372468
O -7.864074 -0.717905 -0.686064
H -8.114432 -1.449640 -1.263097
H -8.664631 -0.193553 -0.562666

IXe
E = -1631.748298
O 0.295103 -1.531764 0.356907
V 1.825879 0.000042 0.110904
O 3.361048 -1.551263 -0.100653
O 0.295099 1.531861 0.356843
O 3.361081 1.551300 -0.100806
O -1.807968 -0.000043 1.167491
O -4.019711 0.000005 -0.188820
O -5.528597 -2.348222 -0.485667
O 5.685906 -0.000087 -0.721258
H 3.399127 -2.257540 0.558246
H 4.275610 -1.251371 -0.255977
H 4.275580 1.251256 -0.256186
H 3.399297 2.257528 0.558134
H 0.107046 2.131368 -0.377083
H -0.569427 1.157907 0.650767
H -0.569413 -1.157839 0.650918
H 0.107022 -2.131518 -0.376800
H -2.683639 -0.000046 0.669764
H -2.034418 0.000034 2.105552
H 5.936009 -0.000105 -1.656129
H 6.521953 -0.000020 -0.234706
H -4.586746 -0.787703 -0.288418
H -4.586780 0.787705 -0.288389
H -6.165245 -2.706539 0.144812
H -5.780865 -2.710036 -1.343992
O -5.528643 2.348226 -0.485620
H -5.781949 2.709394 -1.343916
H -6.164935 2.706457 0.145274

iIXa
E = -1631.798859
O -1.315653 -0.741757 1.383095
V 0.488927 -0.495667 0.321003
O 0.180037 1.505818 0.438008
O -0.109116 -1.653178 -0.985315
O 2.263699 -0.066879 -0.751170
O -2.714832 -1.986056 -0.583139
O -3.636588 0.635706 -0.930830
O 4.254356 -1.296733 0.499982
O -2.355153 1.835400 1.052824
O 2.181094 2.859765 -0.889105
H -1.952992 -1.330317 0.917261
H -1.807200 0.073564 1.600353
H 0.749142 2.174716 0.005288
H -1.799126 -2.052657 -0.956725
H -0.727695 1.856428 0.645174
H 0.471226 -1.845147 -1.734269
H 2.497983 0.858203 -0.932255
H 3.066403 -0.518330 -0.378291
H 2.062971 3.318783 -1.732386
H 2.814973 3.396524 -0.392912
H -3.490091 -0.332324 -0.942022
H -3.130739 -2.852218 -0.669997
H 4.958946 -1.872323 0.176938
H 3.838610 -1.760322 1.240448
H -4.439619 0.811397 -1.433472
H -2.833270 2.491015 1.574200
H -2.953074 1.530278 0.318967
H 1.445485 -1.283627 1.435846

iIXb
E = -1631.798034
O 0.337112 -1.299614 0.004795
V -0.672343 0.423439 -0.155837
O -2.337211 -0.681729 -0.971787
O 1.030346 1.372981 0.620877
O -1.806223 1.237115 1.041472
O 2.740414 -0.815481 1.103817
O 5.038087 -1.199662 -0.268396
O 1.694463 3.416402 -0.964178
O -1.220009 -3.357827 -0.388542
O -4.251502 0.296221 0.445964
H 1.238050 -1.355016 0.402383
H -0.091480 -2.188621 -0.128531
H -3.179055 -0.434839 -0.466707
H 1.805615 0.814084 0.823138
H -2.492567 -0.408019 -1.886900
H 1.334113 2.164088 0.107356
H -3.580578 0.808820 0.948856
H -1.425021 1.862696 1.672633
H -4.860443 -0.100518 1.079804
H 3.583690 -0.966383 0.612496
H 2.929187 -1.040814 2.023804
H 1.090442 3.409987 -1.719167
H 1.835931 4.344785 -0.739772
H 5.414040 -2.042473 -0.551018
H 5.732830 -0.541192 -0.392588
H -1.957912 -2.883009 -0.800125
H -1.164023 -4.223734 -0.810174
H -0.552749 1.293805 -1.583405

iIXc
E = -1631.796709
O -2.071847 -1.545784 -1.153587
V -0.658715 -0.139079 -0.402969
O 0.842145 -1.622963 -0.433233
O -1.994688 1.106034 -0.052878
O 0.838012 1.101555 0.451464
O -3.733621 -0.663411 0.776970
O -1.143984 -1.211688 1.565454
O 3.104310 -0.176044 -0.627160
O 5.465079 -0.595900 0.646343
O -0.153818 3.627089 -0.052975
H -2.926053 -1.438937 -0.677241
H -2.248449 -1.460574 -2.099692
H 1.783442 -1.341897 -0.495960
H -3.379659 0.233306 0.543550
H 0.770802 -2.481501 -0.000023
H -1.789128 2.050179 -0.016018
H 1.733665 0.900128 0.113465
H 0.657979 2.053667 0.299518
H 3.970494 -0.317224 -0.177661
H 3.312719 0.029955 -1.547738
H -2.120067 -1.175170 1.647843
H -4.658079 -0.577978 1.036178
H -0.135223 4.386560 0.543843
H -0.099214 3.995416 -0.944879
H -0.789833 -0.831727 2.379782
H 5.994704 0.070395 1.101432
H 6.038055 -1.365549 0.541168
H -0.178424 0.318678 -1.942466

iIXd
E = -1631.796120
O -0.475105 1.093354 0.686979

V 0.715860 -0.468968 0.149438
O 2.370253 0.758744 0.524497
O -1.085708 -1.518129 -0.253374
O 1.444709 -1.003195 -1.447991
O -3.044538 0.451504 0.431879
O -4.631641 1.428073 -1.524499
O -0.875341 -3.690637 1.268134
O 1.077516 3.283331 1.311278
O 3.839362 0.292128 -1.564077
H -1.463810 1.058406 0.656657
H -0.134051 1.980064 0.923635
H 3.090959 0.675463 -0.167863
H -1.923912 -1.051801 -0.079652
H 2.266954 1.690868 0.769085
H -1.120180 -2.388662 0.220168
H 3.135503 -0.338940 -1.828313
H 0.942836 -1.613765 -2.004836
H 4.679846 -0.178154 -1.618269
H -3.639594 0.821488 -0.264437
H -3.602110 0.303794 1.206433
H -0.902986 -4.639126 1.090845
H -0.002526 -3.504263 1.645794
H -5.092903 2.275555 -1.549282
H -5.040910 0.881087 -2.206650
H 1.181006 3.589262 2.223085
H 1.148950 4.078104 0.764784
H 1.056084 -1.614499 1.319130

H2O
E = -76.423418
O 0.000000 0.000000 0.117802
H -0.000000 0.764237 -0.471206
H -0.000000 -0.764237 -0.471206

H
E =
H 0.000000 0.000000 0.000000

H2
E = -1.164102
H 0.000000 -0.000000 0.380433
H 0.000000 -0.000000 -0.380433

V+(H2O)7
E = -1478.878329
O -2.123928 -1.556000 0.320856
V -0.620010 -0.000051 -0.034483
O -2.123656 1.556188 0.321324
O 0.901576 1.517798 -0.396507
O 0.901986 -1.517082 -0.396797
O 3.175027 0.000085 -0.859025
O 5.152405 -0.000516 0.994611
H -2.027442 -2.136033 1.088752
H -3.051378 -1.253416 0.306363
H 1.796102 -1.181238 -0.621527
H -2.026309 2.138025 1.087737
H 0.721614 -2.270710 -0.972879
H -3.051199 1.253912 0.308404
H 1.795740 1.181754 -0.620651
H 0.721436 2.270781 -0.973514
H 3.887124 -0.000125 -0.175809
H 3.633825 0.000581 -1.708842
H 5.555550 0.771244 1.410657
H 5.555019 -0.772284 1.411156
O -4.517574 -0.000431 0.066291
H -5.274321 -0.002108 0.669186
H -4.902221 0.000447 -0.821949

HVOH+(H2O)6
E = -1478.917497
V -0.151336 -0.892274 -0.380030
H -0.450864 -2.168297 -1.412129
O 0.706536 -1.329401 1.186140
H 0.202288 -1.647546 1.947614
O 1.690143 -0.527212 -1.337583
H 2.005462 -1.212988 -1.940826
H 2.414912 -0.303624 -0.707363
O 2.860982 0.278958 0.923261
H 3.704943 0.189818 1.382859
H 2.225862 -0.352981 1.337319
O 1.316386 2.522944 0.087335
H 2.008308 1.944615 0.465064
H 1.763424 3.247942 -0.365926
O -0.669756 1.040727 -0.668393
H 0.063657 1.710637 -0.493784
H -1.521426 1.391854 -0.344594
O -3.307929 1.321854 0.315701
H -4.008282 1.559615 -0.308775
H -3.530571 1.785834 1.135484
O -2.106491 -1.202707 0.427430
H -2.565975 -1.997522 0.125557
H -2.749987 -0.466348 0.449051

VOH+(H2O)4
E = -1325.454518
O 1.996174 0.884367 -0.050629
V 0.099944 -0.180795 -0.065913
O -0.633408 1.233297 1.457863
O -1.123811 1.181730 -1.318768
O 1.366308 -1.571134 -0.077907
O -1.697660 -1.379443 0.177755
H 2.394525 1.475141 -0.702235
H 2.498868 0.041644 -0.066126
H -1.448632 2.022260 -0.966108
H -1.072533 1.278352 -2.278540
H 1.375266 -2.529400 0.000301
H -1.598119 -2.322235 0.369384
H -2.468377 -1.295673 -0.400160
H 0.045605 1.733201 1.933578
H -1.286141 0.964447 2.119406

VOH+(H2O)5
E = -1401.904594
O 1.359888 1.417852 -0.935355
V 0.020338 -0.019943 0.027738
O 1.708073 -0.227950 1.453787
O -1.688039 0.130658 -1.043249
O 0.933698 -1.634251 -1.124540
O -0.975961 1.818499 0.681867
O -1.388483 -1.464655 0.875944
H 1.734170 1.265550 -1.813668
H 1.011335 2.321344 -0.933511
H 0.323709 -2.167742 -1.653082
H -1.679159 1.553029 0.028421
H 1.566200 -2.251841 -0.733040
H -1.395678 2.097168 1.504693
H -1.854695 -1.517740 1.718834
H -2.023886 -1.148621 0.182515
H -1.859014 0.170516 -1.988273
H 1.685753 -0.599047 2.344856
H 2.430082 0.414849 1.436638

VOH+(H2O)6
E = -1478.352832
O 1.872588 -0.208401 -1.536841
V 0.364853 0.015859 0.035080
O -1.052480 0.492730 1.634484
O -0.838769 -1.558134 -0.494943
O 1.610212 1.675158 0.800369
O -0.778685 1.382227 -1.207542
O 1.290536 -1.743798 0.948918
O -2.867738 -0.023751 -0.237475
H 1.663005 0.009634 -2.455152
H 2.391440 -1.024547 -1.554855
H -0.745626 2.344791 -1.143977
H 1.533770 -2.020590 1.840417
H -1.721821 1.112395 -1.113156
H 0.503848 -2.258619 0.647453
H -1.083444 0.019053 2.474743
H -1.942600 0.377786 1.216944

H 2.526610 1.856883 0.557038
H 1.415002 2.189222 1.593931
H -0.774395 -1.994301 -1.352009
H -3.786306 -0.284729 -0.368485
H -2.256417 -0.819979 -0.405486

VOH+(H2O)7
E = -1554.797986
O 0.528149 -2.176145 -0.124675
V 0.499913 0.000749 0.043714
O 2.115308 0.002242 1.539103
O 0.521171 2.177312 -0.127388
O -1.142652 -0.001318 1.318334
O 2.138808 0.002539 -1.440445
O -0.994983 -0.003493 -1.543836
O -2.101010 2.045935 0.011867
O -2.095655 -2.049639 0.011090
H -0.415582 -2.472242 -0.066286
H 1.042105 -2.748432 0.457449
H -1.580492 -0.774635 -1.411107
H 3.053875 0.005882 1.311638
H -1.581801 0.767488 -1.418533
H 2.050585 0.005782 2.501823
H 1.033306 2.753273 0.452668
H -0.424065 2.469280 -0.068482
H 2.174812 -0.770306 -2.021670
H 2.173653 0.774649 -2.022695
H -1.078396 -0.001432 2.278880
H -2.891091 2.550456 0.235247
H -1.961812 1.319410 0.691628
H -2.887994 -2.552543 0.230062
H -1.958199 -1.323317 0.691566

VOH+(H2O)8
E = -1631.238884
O -0.280123 -2.092908 0.316808
V 0.726333 -0.168140 0.098232
O -0.578289 0.248111 -1.574035
O 2.192257 -0.675949 1.663859
O 1.834589 1.615985 -0.525166
O -0.542054 0.948824 1.301504
O 2.104089 -1.299421 -1.217651
O -2.598838 -1.499971 -0.848064
O -0.532334 2.808517 -0.537855
O -3.088540 0.287060 0.961837
H -1.192911 -2.083304 -0.071555
H -0.348959 -2.528277 1.175066
H -1.404908 -0.277575 -1.583324
H 2.983377 -1.199507 1.483540
H -0.836872 1.190866 -1.555856
H 2.190400 -0.467601 2.605766
H 2.580161 1.981417 -0.034272
H 1.165232 2.337797 -0.614945
H 1.783446 -2.156469 -1.531151
H 2.422793 -0.825938 -1.998843
H -0.298033 1.186871 2.202228
H -2.164676 0.563406 1.230650
H -1.001754 3.648936 -0.493712
H -0.691186 2.293646 0.309569
H -3.307376 -1.989179 -1.281842
H -3.003412 -0.898262 -0.157556
H -3.667037 0.368429 1.726999

V(OH)2+(H2O)3
E = -1324.890223
O -0.464250 -0.976378 1.508120
V 0.000295 -0.084821 0.000062
O 0.465044 -0.976741 -1.507825
O 2.123476 0.046307 0.227293
O -2.123330 0.044667 -0.227558
O -0.001350 1.985726 -0.000336
H 0.115364 -1.445383 2.121995
H 2.504271 -0.479899 -0.496204
H -2.637395 -0.141649 -1.025275
H 2.637760 -0.139535 1.024994
H -2.503450 -0.482036 0.495919
H 0.783727 2.549618 0.044773
H -0.115676 -1.446215 -2.120325
H -0.788103 2.547333 -0.044866

V(OH)2+(H2O)4
E = -1401.335139
O -0.973003 1.688607 -0.956177
V -0.396430 -0.008321 0.072834
O -2.320236 -0.814358 -0.476690
O -1.055985 -0.297619 1.735083
O 1.308399 1.118893 0.539564
O 3.221138 -0.423155 -0.156337
O 0.657698 -1.278697 -0.733665
H -0.422785 2.482924 -1.011828
H -1.828211 1.859813 -1.374087
H 2.169574 0.645089 0.285076
H -2.735808 -1.088414 0.358207
H 1.438575 1.509315 1.413459
H -2.538135 -1.478333 -1.144624
H -0.571461 -0.214640 2.566909
H 2.606972 -1.050057 -0.583879
H 3.985052 -0.306389 -0.734267
H 0.310023 -2.117301 -1.064360

V(OH)2+(H2O)5
E = -1477.780428
O 0.967674 -1.165082 -0.883872
V -0.103815 0.392636 -0.093421
O -1.705584 -0.352487 -1.234156
O 0.010645 2.071235 -0.780532
O -1.068471 0.108729 1.445496
O 3.473801 -1.196322 0.148441
O -3.308752 -1.166170 0.589890
O 1.684321 0.968857 0.947200
H 1.896344 -1.381297 -0.638870
H 0.582489 -1.851789 -1.443898
H -2.480733 -0.669129 -0.665748
H -0.680657 2.523361 -1.281031
H -2.058984 0.097797 -2.010859
H 1.864400 1.893528 0.714993
H -2.751785 -0.721681 1.262074
H -0.687756 0.363906 2.296898
H -4.232811 -0.989446 0.802941
H 4.254767 -1.065848 -0.408819
H 3.742744 -1.845622 0.814561
H 2.510659 0.465504 0.846702

V(OH)2+(H2O)6
E = -1554.223167
O -0.345855 -1.474210 1.788127
V -0.234808 -0.531387 0.231892
O 1.182345 0.898790 0.010036
O 1.274484 -1.992828 -0.221504
O -1.284087 -1.032859 -1.201464
O -3.580522 0.345488 -0.872484
O -1.628835 0.986773 0.672218
O 3.567826 -0.382946 -0.718329
O 1.081283 3.538915 0.109218
H -1.059375 -1.391684 2.433508
H 2.194616 -1.712621 -0.380562
H -2.970266 -0.290772 -1.304966
H 1.272999 -2.605929 0.530539
H -1.022716 -1.808531 -1.715676
H 2.096480 0.675489 -0.253742
H 1.078363 1.885331 0.071997
H 4.349208 -0.366994 -0.147716
H 3.912439 -0.335073 -1.621443
H -2.470689 0.901946 0.120077
H 0.559777 4.087221 -0.490515
H 1.269411 4.085188 0.882772
H -1.893145 1.176038 1.580748
H -4.049620 0.825310 -1.565076

V(OH)2+(H2O)7

E = -1630.664045
O -1.225273 0.975283 -0.900255
V 0.060203 -0.523540 0.034835
O 1.106791 1.297659 0.589000
O -1.058840 -1.793767 -0.743177
O 1.366893 -1.424403 1.021463
O -1.332348 -0.310794 1.649250
O 1.397585 -0.622841 -1.631715
O 3.422314 -0.103020 0.062076
O -3.320647 -0.673481 -0.115521
O -0.450790 3.567762 -0.023327
H -1.183004 1.912805 -0.636166
H -2.158189 0.685085 -0.852621
H 0.825307 2.211575 0.410449
H 2.077732 1.219954 0.524519
H -2.729130 -1.364940 -0.507762
H 1.170222 -2.029026 1.745729
H 1.236564 -0.116521 -2.437433
H 2.322381 -0.454312 -1.337020
H -1.213004 -0.767820 2.490618
H -2.244044 -0.497193 1.323578
H 2.949871 -0.775282 0.614589
H -0.273443 4.263461 -0.671353
H -4.238168 -0.947820 -0.227696
H 4.371908 -0.227919 0.174045
H -0.850895 4.019872 0.732493
H -0.694239 -2.389678 -1.409522

HV(OH)2+(H2O)7
E = -1631.199186
O 0.954862 -1.954109 -0.206498
V -0.402085 -0.712964 -0.310457
O 0.746216 0.708702 -1.454033
O -1.777665 -1.533112 0.351691
O 0.624454 0.335294 1.239091
O -1.687040 1.030869 -0.453504
O -4.197553 -0.043661 0.254985
O 2.957369 -1.023280 1.244393
O -0.022040 3.034309 0.703992
O 3.282343 0.833160 -0.753121
H 2.368177 -1.650594 0.754372
H 0.940274 -2.675198 -0.849402
H 1.512582 -0.001309 1.498236
H -4.827120 -0.215015 -0.459169
H 0.629332 1.310383 1.226675
H -4.737205 0.084741 1.046998
H -2.613762 0.935706 -0.166372
H -1.325269 1.872871 -0.121104
H -2.713846 -1.258719 0.386339
H 0.611945 0.600755 -2.403470
H 1.733191 0.719065 -1.285099
H -0.236615 3.710441 1.361047
H 0.546320 3.474024 0.056337
H 3.453603 -1.523910 1.902856
H 4.065893 0.911727 -1.309682
H 3.475475 0.157265 -0.070785
H -0.682597 -1.159429 -1.843222

IR frequencies (in cm^{-1}, without shift) and intensities (KM/mole) for optimized structures

IIIa
29.2116 6.63
100.4123 27.9613
115.6578 9.0529
127.5987 0.7713
154.9182 101.0679
256.4148 0.8872
294.7278 69.7465
298.7716 0.0317
321.5702 23.7717
327.5694 157.8457
378.3872 83.5792
383.6366 135.821
471.6229 1.6006
497.5826 6.6675
500.6928 60.2387
1620.7903 158.5086
1626.4813 84.0752
1629.1639 117.9509
3742.5625 40.9156
3747.7323 61.9026
3769.9117 57.4104
3831.2512 138.508
3836.5109 177.7459
3871.9189 166.1723

IVa
44.4627 18.2648
48.6768 109.8995
61.1796 10.3131
72.0393 0.0327
94.0252 2.1339
120.9735 2.0443
135.6046 22.9128
152.6394 147.0518
171.3094 0.9112
217.0438 156.071
261.265 0.0692
268.7703 1.4151
285.5793 3.6592
320.4889 131.5965
331.2421 15.814
337.8662 37.1394
365.5608 172.8243
434.9625 0.0166
449.9725 0.0048
475.7373 0.0061
484.9311 48.9199
1615.4008 220.4813
1616.6203 45.151
1621.2947 131.8713
1625.0472 97.8528
3741.77 12.7396
3743.3627 12.5991
3757.5923 9.5001
3783.3327 52.3216
3835.5795 71.8137
3837.8748 136.7695
3852.0875 151.3951
3879.8655 134.8443

iIVa
87.097 0.1598
108.3064 3.2887
137.1009 6.1011
144.2954 7.0902
160.8182 18.435
213.4439 12.3564
244.3739 19.1611
320.9888 0.403
350.9581 146.9492
353.1423 30.2364
376.2187 9.6924
378.3559 82.8932
416.4697 203.2819
435.3377 163.6432
476.8875 120.2836
519.3671 100.4495
553.8967 149.985
566.3846 58.2565
579.2894 23.1244
634.4703 60.9039
691.6552 109.8973
760.1307 172.994
1620.7659 141.3335
1622.5389 11.6725
1625.1262 177.8241
1769.6226 181.9254
3716.7458 91.6611
3744.1504 95.8676
3749.2419 119.1102
3805.3278 104.0659
3817.4222 166.3532
3834.5071 161.8393
3843.816 193.5232

Va
32.9237 28.2167
45.2402 3.882
78.2181 0.3473
81.1911 0.2342
81.5809 16.3737
95.7964 2.1454
135.1324 4.2821
144.2206 32.1887
145.5624 2.7033
204.8959 1.5774
209.7341 34.4339
217.3749 20.4595
233.4548 314.7104
257.262 101.6293
276.8767 0.1047
288.3653 5.5347
290.1779 1.5139
316.8508 25.6155
355.3681 43.6392
355.6662 11.0391
433.0625 110.9896
441.8084 0.0736
444.1005 2.2618
556.8325 115.6597
616.7921 98.3348
619.7621 30.9262
672.1362 90.2829
1606.5089 92.2991
1609.8244 91.1149
1610.97 210.1004
1617.4893 101.7377
1655.6766 4.2414
3610.4294 71.5625
3641.4229 804.7842
3737.7826 3.5817
3738.21 5.2395
3764.5485 15.5152
3833.9546 1.2542
3835.4902 213.4392

3854.5208 196.3706
3856.2098 92.0926
3857.4376 124.9999

Vb
21.6662 0.28
35.8649 11.4231
45.3816 14.8264
60.5851 3.5889
81.8556 2.2224
87.0322 6.3781
103.5745 3.3766
129.0041 83.894
135.1881 25.9489
144.4401 43.0649
144.7843 108.0253
208.0118 35.1177
220.7977 18.2062
273.3446 50.0383
279.3072 51.9767
291.6335 54.6325
296.9954 26.7893
318.4296 200.9197
333.0714 65.4282
351.226 71.4166
354.389 127.8514
375.6288 13.8073
459.213 8.3538
480.7629 1.9727
499.4348 37.1697
570.3715 23.977
841.763 90.6142
1612.2047 137.4586
1621.5211 162.4664
1623.2223 55.974
1623.9774 31.9953
1644.5413 98.5472
3382.6882 1115.2107
3735.1923 7.2919
3749.6183 15.7497
3755.5025 19.9802
3792.6414 34.8981
3818.5699 74.0355
3831.2887 114.3775
3844.5228 115.6927
3850.3409 111.0474
3894.9577 117.3446

Vc
18.7748 2.7385
32.7532 4.7276
44.8587 1.5019
51.2566 1.911
66.096 2.1835
94.4042 4.3704
102.5563 12.1973
123.9884 52.6301
130.4916 17.9298
138.5002 40.8467
145.0484 123.8932
204.7683 212.4798
231.0696 28.5659
245.0419 27.9977
271.0396 2.814
297.8225 4.8116
308.3309 11.3953
322.6747 103.8551
337.2626 86.2302
363.6326 153.7401
387.0562 62.5897
438.5907 16.7959
468.3519 1.2188
618.8253 7.3703
630.1283 29.8597
823.0012 68.6093
884.4896 84.0305
1616.2812 107.2775
1618.5624 175.2674
1619.1637 63.3799
1642.3329 48.0606
1649.868 25.2816
3287.0869 1347.831
3389.5181 1352.4732
3745.9884 31.5181
3790.452 35.4664
3791.5513 39.53
3813.9911 95.8137
3828.7162 88.8254
3838.8498 126.7149
3892.501 118.2542
3894.6214 127.3752

iVa
38.3333 1.2881
49.7487 1.2891
92.7497 5.4058
113.4515 4.5279
128.9751 5.3963
142.5947 5.8748
152.0293 5.3223
170.4208 16.244
185.6586 179.5454
212.7936 4.8591
247.442 94.7223
268.1313 38.607
316.1228 0.1836
346.876 7.6371
373.8642 17.1035
396.473 66.08
413.8249 258.2565
432.0766 151.7773
459.8412 76.3812
473.6199 100.1538
516.4997 113.415
564.9828 93.5939
581.3717 4.5567
637.8671 32.0216
691.3742 112.6218
711.2859 69.7966
752.199 174.9349
961.4099 99.9182
1614.1757 123.608
1618.5005 21.0648
1618.9856 168.1235
1649.1354 40.0594
1755.0419 207.6862
3111.8835 1957.4626
3717.6891 78.1272
3748.2457 78.7949
3786.3997 49.4949
3815.3307 95.0964
3817.1684 127.4316
3829.0125 152.1785
3840.2396 153.3302
3887.2559 144.1114

iVb
43.9311 8.8577
81.7001 0.3322
113.8155 1.0327
118.811 12.0222
144.0091 3.9438
152.3304 1.6531
201.3793 59.1476
222.1512 9.6143
258.7883 82.7451
310.9854 50.6202
322.1758 109.8984
325.8906 71.8592
354.504 8.2128

375.0091 36.9845
392.8141 20.9835
403.0573 228.92
411.0561 68.8154
428.8181 77.0437
454.1063 126.2364
504.2976 11.9609
530.2392 126.142
546.6208 87.8144
565.128 58.746
630.0512 111.5075
699.0425 90.3273
719.7776 220.1977
752.6026 64.3111
1022.6555 111.0151
1585.8614 92.6458
1613.604 109.786
1617.4368 132.6125
1658.0016 59.4785
1766.3108 179.3265
2946.0426 1414.5904
3667.9815 201.0837
3748.6372 94.0194
3751.8851 113.206
3804.7599 91.7641
3810.6042 128.9756
3841.8322 151.1002
3851.424 185.8704
3862.3842 188.4397

iVc
45.1119 7.4196
46.9501 7.5401
92.569 53.0734
106.2222 9.4026
140.1239 2.2818
155.346 11.0546
171.1927 15.4665
197.0965 28.3468
223.1678 95.1071
254.7795 20.4217
284.6533 26.2249
298.5148 93.7013
319.2189 89.7448
331.517 242.2255
347.2029 46.1416
368.9169 15.5501
382.8287 27.5099
411.9729 297.0964
437.2261 6.8173
484.2434 98.9121
505.5352 78.8891
534.5301 114.2556
565.2844 31.6343
605.5337 28.0313
688.6908 195.2855
697.1692 139.0004
722.7203 47.0219
1004.2316 115.8521
1577.0744 97.9214
1607.8119 127.716
1616.2718 119.2332
1646.3262 74.3919
1755.4375 177.8988
2975.3818 1393.527
3701.3635 121.3952
3756.0221 94.394
3762.716 107.1008
3797.2766 123.8363
3817.4332 141.4961
3852.3884 157.4435
3862.7875 170.4785
3869.1162 192.7617

VIa
23.7803 1.6819
36.2283 1.1314
55.668 11.6028
69.3366 0.2602
79.1236 0.1406
91.5242 0.005
93.9687 3.042
98.0657 11.1213
108.1642 32.7231
127.3656 120.0966
143.522 59.7691
153.6604 45.9929
174.8071 1.847
205.7425 64.2545
207.0362 122.7595
229.215 108.8974
232.3448 234.6862
278.8178 1.4162
288.6414 6.706
293.8644 30.7402
303.0003 31.6125
307.1226 9.8339
358.7482 43.5765
363.0474 8.6022
392.314 17.9595
427.723 3.6858
436.0404 3.3797
436.1651 0.4862
591.8377 77.9827
656.329 125.0418
660.9725 10.2611
777.273 78.1911
903.2662 94.7481
1603.1706 81.1385
1607.5357 202.0287
1610.3229 91.0929
1621.8556 94.1909
1628.3804 36.7709
1670.642 13.1255
3398.8988 1582.1759
3482.6994 144.116
3547.5435 760.9129
3735.71 8.2755
3736.0779 1.411
3794.4284 32.1325
3833.2941 0.0287
3834.1171 63.9927
3834.7469 223.415
3855.1043 171.7601
3856.0068 61.8886
3896.8222 119.2295

VIb
11.8195 0.2839
30.1653 6.9128
39.6496 7.8205
67.5391 5.5572
77.1071 7.8788
83.9911 1.6781
93.118 2.2503
115.0158 12.9161
126.9412 12.5495
131.955 8.1634
146.6623 3.137
159.8675 56.2277
209.7195 23.8758
210.901 48.0604
217.424 18.9233
226.8052 87.942
246.6378 63.0881
264.4743 199.6587
277.9527 6.6119
280.3146 5.3879
301.6642 106.5802
311.3797 4.8498

335.3601 76.034
350.1947 14.5621
376.1232 49.0046
426.1438 116.0321
445.6556 4.3922
537.0726 78.7887
592.1063 136.9569
601.4648 14.6198
626.4354 41.8101
680.1253 64.2015
739.8513 71.6463
1602.6884 141.2648
1605.7475 81.459
1615.8261 125.8831
1622.8813 98.8092
1651.0203 10.9336
1653.2152 61.2325
3421.9971 897.4996
3619.3969 69.5829
3647.2058 681.2575
3725.6285 10.3824
3765.1274 12.2985
3785.7309 23.1531
3818.9413 68.2359
3824.1495 105.7356
3846.3932 146.3859
3851.8945 131.7843
3859.2292 117.9671
3886.9523 107.1743

iVIa
32.1944 6.8225
55.2065 1.5771
93.495 0.4553
93.9865 0.3114
130.5842 2.5289
138.6049 2.778
155.7363 1.0915
165.9196 1.3104
218.2973 77.2433
240.0969 19.1781
254.2858 34.8168
278.2301 56.9201
298.0415 23.3485
314.2148 74.2958
328.76 84.8735
336.7782 19.8731
363.1573 64.1237
394.8108 41.0072
403.5772 23.4222
421.4414 41.4927
446.5793 210.5184
467.4763 104.3309
483.5944 69.0686
522.9733 17.4871
553.4829 111.2266
588.3446 96.0895
596.7959 45.6064
663.6662 175.153
695.0132 80.3332
717.0352 78.4774
736.0111 166.985
779.5661 113.6259
794.9983 192.4302
1021.3076 106.3065
1592.6977 89.4297
1599.672 110.7595
1622.8289 112.6956
1655.0699 29.2853
1664.4369 51.3179
1757.8149 202.9634
2972.8025 1335.4955
3450.0437 734.9057
3606.6225 370.8213
3639.4705 482.1564
3756.3026 36.0231
3794.6512 88.0034
3817.2339 120.3338
3819.2987 232.7647
3825.0487 142.8995
3845.2693 146.7256
3860.2157 163.4348

VIIIa
9.4753 2.3495
27.1885 2.721
27.8463 0.0411
36.0862 0.2255
43.617 4.5563
72.2596 0.1306
76.0583 3.1388
81.1432 0.6452
88.6541 2.7146
93.7257 0.9765
94.4083 4.6185
122.0332 145.7625
129.6427 65.8024
137.4448 63.1863
145.0539 208.3711
157.8576 0.1281
173.9422 0.2146
187.5461 23.0832
202.3764 61.6703
231.9433 0.1193
253.4314 5.4893
280.5952 96.0711
286.0396 9.2735
286.1451 0.5452
289.0041 39.8236
300.5241 7.2092
303.1256 219.4155
304.9151 8.7834
332.1645 11.8087
355.2419 58.8026
355.5837 4.2002
369.4585 4.3237
402.5385 103.9606
427.7815 25.2244
439.7012 74.2234
542.8374 57.225
572.5248 0.487
598.5847 78.3302
620.8875 85.7744
661.1363 4.021
702.3528 162.1961
799.1437 2.1322
806.4235 64.4282
856.8003 105.8654
896.7565 137.3859
1621.3514 120.6743
1621.5273 52.8451
1623.1274 39.2208
1625.8034 27.9865
1634.8592 105.9003
1637.7703 36.6332
1658.2811 35.73
1665.4285 32.2137
3407.3906 1657.285
3425.3488 1309.3684
3484.9377 31.5375
3490.4487 286.0104
3544.6982 839.1871
3547.1253 123.5652
3794.598 31.3795
3794.8152 30.4894
3800.7544 29.4968
3803.0769 151.1879
3819.6574 72.0914
3820.8318 128.8235
3825.2099 79.6748

3835.214 91.9667
3897.5332 104.4824
3897.9432 125.3426

VIIIb
13.7134 2.9293
25.783 2.8862
31.1475 3.2643
44.1216 12.8609
59.1049 2.0242
69.1118 0.6621
72.2046 7.0612
78.9808 3.5795
90.0108 2.7855
97.5833 2.1899
110.5561 199.8217
126.4165 19.4421
128.2696 2.4677
137.5823 1.7951
149.7445 0.4643
181.8792 2.9105
189.4464 0.353
197.5695 46.5245
205.3258 21.0627
212.0168 12.1999
237.7161 5.2642
260.4809 23.5424
272.3018 13.8402
282.721 39.171
289.884 9.3472
296.6174 21.0575
314.0871 10.3185
339.6392 54.8139
359.8158 47.798
362.1809 36.0293
372.0591 80.8334
394.6107 89.0551
411.7008 185.6053
424.5705 41.3051
529.2245 33.8913
545.9872 169.3516
557.6474 113.3488
575.2964 89.9132
585.7591 124.1586
610.292 66.9473
684.1088 65.2416
727.6396 34.5493
747.4108 54.9116
763.1874 45.5388
875.5435 108.0526
1583.2508 61.672
1595.2085 86.5307
1620.6155 111.5176
1622.5515 60.2065
1630.8665 34.636
1631.3442 73.2315
1639.0138 39.797
1658.0032 25.6495
3443.668 1322.2991
3506.8329 210.3753
3596.1201 321.1838
3598.1906 228.7823
3636.9865 100.0042
3729.7699 413.2669
3740.5109 495.5781
3762.9795 19.3048
3773.504 48.1112
3794.5434 128.3912
3795.3964 23.6174
3811.5858 97.8509
3836.1523 83.841
3858.481 95.791
3865.8243 104.0666
3898.9262 111.8028

VIIIc
15.6818 0.0
40.7391 30.4339
50.0973 1.3016
50.1415 1.2957
70.1776 4.705
70.2071 4.7239
70.8374 0.0057
91.0438 0.0
100.5176 0.0
121.9572 0.0459
127.7316 0.0034
139.1173 1.0595
139.1723 1.0418
146.6753 1.9677
193.0466 0.0002
199.0149 12.1853
199.1654 11.8162
206.5241 28.7199
206.6024 29.3223
222.0939 0.0007
232.7061 22.4702
258.6308 0.0039
269.9495 0.012
270.5109 1.5717
270.6685 1.596
296.1198 0.0
321.9127 15.639
362.4585 51.1282
362.4904 51.1817
384.9687 0.0026
403.2484 187.7319
403.2821 187.9124
411.9715 0.002
525.627 0.0012
570.9699 5.1872
571.1263 4.0671
578.5068 0.0646
588.4231 453.7316
588.5951 453.0993
599.6035 0.0773
601.3485 284.9633
663.1859 0.0171
725.5149 34.3575
725.9457 34.1428
751.9457 0.0017
1585.6973 0.002
1596.3899 119.0549
1596.4492 119.2135
1607.5073 38.3718
1625.9495 0.0004
1632.1254 102.3975
1632.1465 102.3892
1645.0263 3.1148
3626.2811 0.0609
3632.3277 64.0582
3632.6293 63.6566
3641.7106 45.656
3712.8508 0.1915
3724.5195 956.2129
3724.7906 953.5902
3731.1184 0.4107
3764.8352 1.4399
3767.9646 57.4545
3767.992 58.308
3771.2218 0.0048
3862.7986 0.085
3862.9341 4.3818
3862.9447 5.4493
3863.1844 379.9498

VIIId
9.8501 2.93
13.1212 1.1812
21.4715 1.4262

33.1883 3.6325
47.221 0.8767
67.1176 13.2084
70.3196 52.5483
75.8142 3.5857
90.7212 3.3103
95.3352 0.9954
129.7333 27.746
132.3438 1.5072
137.524 0.5492
146.8606 30.7933
159.6964 202.8751
178.5967 11.0236
190.1916 18.4571
191.9406 38.4702
209.45 3.0274
223.4754 14.3328
246.4048 14.2104
246.9324 83.7932
263.9353 116.5299
279.7434 17.0274
285.7413 8.0746
302.0584 1.8796
326.8396 107.8908
336.6364 10.4315
345.9999 13.3935
357.4787 82.6422
364.8151 14.455
398.5559 80.8869
408.4308 169.4946
453.4709 9.7515
474.1314 23.6946
518.1149 40.6788
529.8041 60.8483
589.0874 0.2577
623.5638 207.6717
649.6826 105.5322
697.4958 28.9303
715.2452 19.9089
737.9024 115.8395
829.9895 44.9133
991.561 107.1916
1617.2449 97.3299
1618.0677 25.3482
1623.0242 142.0162
1625.0246 52.1482
1637.1822 77.0512
1639.7576 48.8056
1642.8224 13.4929
1672.9013 24.1467
3208.2868 2170.2034
3421.3937 185.2777
3490.5114 976.79
3531.25 653.394
3601.96 104.6035
3622.992 342.0572
3756.819 8.5368
3793.2816 23.8331
3795.2961 24.6876
3797.9591 207.8771
3829.2921 90.1349
3830.8087 87.8206
3839.0054 85.4249
3853.1736 101.7363
3874.0243 99.6624
3899.5019 108.3692

iVIIIa
26.9415 1.9044
37.6045 5.1718
49.1118 6.7924
68.4627 4.3847
79.4691 3.5393
91.6706 0.7654
107.8031 1.1749
135.5309 2.7108
148.1675 6.7193
150.1037 13.8466
157.5442 22.4775
172.2495 4.5699
175.0684 3.0538
204.0898 6.6741
239.0949 82.3777
252.058 34.4205
255.1536 45.1306
257.1698 23.9054
266.1274 74.6278
279.4625 185.5218
301.204 2.9493
309.5171 24.5685
327.3247 37.8381
341.3922 11.5212
354.5282 54.5818
400.3157 125.4973
412.6752 106.8322
431.1155 83.9138
440.478 60.6559
455.8576 35.0428
492.4719 53.328
527.3582 50.5848
548.1148 174.8967
548.9357 13.885
607.7506 32.311
616.6226 65.0739
670.5216 133.0328
684.1006 78.4984
713.1472 83.7487
739.8199 127.7261
746.9787 271.2782
801.5873 215.9188
837.085 57.9909
922.3113 91.8683
944.5055 78.4967
1035.2374 114.6015
1609.7526 98.2931
1616.5976 27.3984
1619.8743 97.5132
1622.4868 179.9378
1633.0172 3.5522
1651.269 122.8343
1668.4412 8.4722
1715.7357 258.3724
3036.4363 1501.6354
3270.6447 1314.1087
3360.1422 321.9015
3453.4874 868.5581
3549.9619 693.5697
3587.033 350.5454
3680.0342 629.813
3767.6947 12.3517
3781.3619 20.5265
3796.4881 72.8135
3833.8524 153.5369
3848.623 128.7695
3856.5256 132.8281
3859.3156 131.9065
3884.7702 124.125

iVIIIb
33.4178 4.2251
46.3757 0.6844
68.5868 0.87
80.9302 1.2214
91.9864 0.4892
104.5001 2.9146
134.0105 1.0281
139.7181 0.1801
147.3341 5.5344
171.2212 19.6797
175.9227 8.0119

180.7237 45.1236
192.9346 19.2906
214.924 17.7116
242.6536 2.6425
257.5297 21.5939
265.9272 95.0893
275.5086 20.6329
290.5549 4.3755
313.1819 36.3404
331.2762 1.9568
344.5762 8.0715
366.9215 113.3032
370.067 74.3846
409.7957 30.2975
423.5319 47.7304
443.7356 20.8485
452.0826 151.1188
476.3448 54.2939
490.6864 40.2043
524.123 27.6228
555.0044 118.0998
574.3161 66.9697
586.5146 77.9694
615.373 9.7114
641.9912 74.5693
682.1347 77.8008
704.2624 103.3431
725.0175 238.6489
749.5081 93.9897
775.9817 347.9771
811.8228 51.5814
834.8739 314.4975
886.037 105.5903
953.2439 30.7672
963.3271 111.0461
1598.0545 56.6625
1616.8736 117.6819
1621.8734 133.4175
1626.9929 47.4461
1647.0053 40.0944
1664.2541 20.1672
1688.6789 14.736
1761.1895 219.4099
3175.5531 1108.8822
3279.3496 605.7946
3326.8342 464.8016
3435.6896 1206.2535
3567.0742 294.3377
3574.7811 140.8269
3613.4261 821.4559
3617.4249 985.0794
3762.4284 24.8627
3806.0107 78.9952
3821.7706 165.2745
3845.4942 124.7544
3848.3554 100.0513
3852.925 138.4821
3867.56 135.1563

iVIIIc
27.451 0.9785
35.7075 1.5383
50.3247 5.4567
65.0205 1.6357
85.3245 5.8683
89.8398 1.5474
124.5228 2.2985
136.2499 8.2247
148.3187 23.3491
153.0926 8.8696
165.546 1.3785
171.9428 50.2055
176.9096 31.1294
188.983 22.8594
241.7295 13.6135
251.4177 136.5098
262.3511 69.9095
267.4413 87.5776
274.2398 107.3862
308.0285 33.3833
312.5015 34.8036
342.8576 0.9536
356.4089 38.2539
367.877 51.8798
372.7731 105.4288
383.4771 43.732
417.8025 27.5061
419.5303 38.5018
442.6668 26.0499
452.4587 31.3191
486.0689 49.3665
525.5461 35.6526
564.5746 73.6187
588.213 65.051
595.5378 52.8592
629.7639 5.3687
635.9562 137.6561
688.5679 161.0737
713.1835 195.9285
750.3118 140.2699
775.2792 227.6626
812.4141 233.3823
860.6251 120.2413
937.2162 70.1698
944.5939 51.9536
954.6359 82.338
1598.3839 47.9747
1608.7981 105.2935
1617.1228 111.6764
1627.9647 112.0826
1656.7126 100.9728
1662.8698 10.4915
1687.6318 11.4806
1755.6876 220.2612
3110.7223 1512.2878
3192.8394 1222.4641
3273.276 655.4641
3349.6086 296.8396
3440.1661 1208.9053
3582.3437 279.7182
3626.1598 717.9058
3781.1924 28.9136
3814.6939 65.3062
3827.3927 97.2679
3834.6649 152.1782
3844.8671 129.3718
3847.0343 105.58
3867.9327 141.2183
3885.2338 133.0444

iVIIId
18.2872 1.0378
28.0212 0.5124
35.9787 0.5399
44.8877 7.3254
55.205 13.8616
70.3193 3.1191
78.9856 5.3268
89.5522 4.6122
101.8321 3.9212
129.1259 6.2152
130.4095 7.4429
148.2014 2.4335
156.2756 14.8707
174.269 2.6619
182.5632 19.9849
194.7616 224.4579
209.341 85.0524
242.8058 33.7296
251.7795 18.6723

278.9724 52.6993
294.7633 7.7858
304.9447 49.7294
319.5748 22.4081
322.4615 76.4175
344.8096 3.5834
363.6329 79.7609
376.5848 99.9463
398.3383 53.4107
406.5529 57.8899
422.8201 173.0965
438.9915 35.2463
466.6683 47.5941
487.3092 42.609
557.1275 25.9483
573.8697 65.0421
593.7376 122.7531
621.5168 49.1478
700.7119 84.8546
720.5318 166.0315
724.806 55.7243
745.3803 348.9989
778.7431 235.2392
829.5168 27.1987
872.1935 112.6536
917.2667 74.2468
964.6459 70.2555
1586.3014 37.8821
1593.1081 188.9589
1607.1554 106.8042
1626.4292 51.5693
1634.8423 52.3931
1650.2157 59.2257
1666.0852 28.2366
1708.0736 245.2248
3246.631 1269.4504
3340.6004 1640.7535
3400.6386 334.4676
3547.0998 782.2392
3579.3524 587.7414
3718.852 511.6778
3735.6521 41.0279
3769.8073 5.1108
3790.816 33.7175
3795.2017 65.5016
3808.4001 184.4376
3829.0954 87.1164
3861.3507 129.2033
3865.3047 150.183
3891.3447 122.5347

IXa
10.165 2.4461
24.2033 0.8125
25.442 4.024
31.738 0.7036
39.6364 0.6416
43.3179 9.326
63.6525 5.0464
69.1412 5.7517
75.6613 4.7594
77.1929 4.3318
84.0116 5.6224
91.9953 5.1824
98.946 1.596
112.8615 197.6465
123.8851 57.2827
130.7217 156.9711
136.3579 11.2431
140.479 15.3886
152.8582 0.9618
181.7764 2.5313
184.3088 4.1447
194.6033 68.0165
206.6029 15.487
250.7491 10.507
256.8058 25.0971
273.1836 23.1908
276.641 10.6649
279.7024 33.8903
286.5736 1.028
292.8697 6.9172
309.5752 34.2394
323.5644 123.2917
356.9381 38.7367
361.3143 25.4096
366.4247 27.4677
372.7321 43.8198
397.8369 161.1788
415.7005 5.9779
422.1616 61.1722
553.2499 17.7507
559.481 121.2428
569.8308 111.5145
578.4876 98.8776
592.3048 175.2963
632.301 59.8804
720.1504 20.4486
743.9317 46.9311
760.6001 52.4491
785.17 39.1382
846.5867 112.5859
870.878 110.9698
1580.439 53.0643
1592.5957 77.5789
1621.0237 110.0722
1621.4414 54.8141
1630.9001 17.1993
1631.5271 76.982
1632.001 53.3174
1653.1896 50.3211
1657.6744 20.7331
3451.9195 1603.501
3459.599 987.2363
3511.8337 28.5953
3514.697 308.9711
3599.341 338.1202
3604.9696 123.5812
3723.9678 386.2573
3738.1429 518.1794
3774.1823 41.7281
3794.9929 29.8964
3795.7116 27.3917
3797.0631 82.684
3805.6619 104.187
3833.9355 83.2967
3836.0767 83.4864
3868.0344 97.5686
3898.45 106.7575
3899.6085 112.7665

IXb
10.0903 4.2082
16.3597 0.9673
26.668 5.0627
30.2762 3.1957
34.3232 0.5467
41.5901 2.6453
51.6787 7.9854
64.433 0.6009
66.8019 12.6031
73.005 7.2925
85.4052 1.1447
96.0325 1.8251
97.3681 20.0748
103.8834 180.2695
115.2273 182.3863
122.0631 45.1474
130.2723 6.0878
133.6809 6.7273

156.9326 0.5613
182.7942 10.194
197.5389 41.1679
200.348 33.7258
201.8542 27.9465
236.9931 3.1732
265.1265 20.0754
268.508 16.398
272.3421 14.7008
280.2994 56.6381
291.3457 17.4354
294.2725 13.1235
312.0507 8.316
329.1636 7.8339
349.752 34.2996
362.6194 63.3717
367.1613 64.0602
377.3112 41.0678
390.9699 149.2328
412.2912 79.5944
418.6571 27.6887
526.6523 44.2819
537.4264 122.205
570.755 112.8897
595.1544 88.0564
606.0697 32.4344
632.9882 172.9722
701.3899 52.6135
747.4614 28.6513
751.3014 37.8438
776.2404 74.7485
857.5 96.0374
870.7137 108.9337
1575.944 51.3623
1599.7802 69.5864
1619.3566 104.0978
1621.5385 32.5707
1622.1037 71.222
1630.7862 74.3359
1632.4474 44.3422
1652.1791 45.2143
1659.8083 39.3709
3455.0644 1214.6974
3477.9171 1251.5839
3508.7223 223.969
3555.5092 133.0277
3589.4323 212.4338
3607.9502 252.0556
3699.912 691.5421
3735.159 272.8048
3762.5592 33.0404
3787.7154 129.9766
3795.901 24.203
3796.5296 24.5013
3812.3376 96.1414
3837.4275 82.4507
3838.905 83.6625
3858.5839 90.067
3899.8034 94.9193
3900.2892 123.269

IXc
6.7256 2.4396
10.3164 0.9616
20.7783 0.7992
25.8713 0.3021
30.1078 1.5618
39.7572 1.1567
47.0159 1.6006
68.1659 5.6873
76.9114 3.8846
87.3804 1.2323
92.0298 18.0034
92.7264 17.5333
97.1183 1.2926
125.6433 60.1753
134.4764 139.1094
138.8876 33.1349
163.3154 6.7936
168.9664 200.9838
180.0483 38.4312
185.2106 19.9119
192.0534 38.1381
193.6271 42.8288
237.9393 8.7631
248.2646 45.1449
260.4493 2.3164
266.6917 12.4416
285.65 9.8314
286.8572 11.9841
292.6069 17.0785
302.9879 0.5253
314.8723 276.9288
329.9987 48.8486
346.0874 18.8527
354.8251 64.3691
359.8464 7.152
385.4382 100.1118
396.17 13.8953
432.3459 23.0563
445.8596 49.7715
488.7765 5.001
562.0137 143.6949
574.3215 69.9537
588.1485 22.7561
615.4731 36.6427
721.6871 73.0892
738.4149 105.9625
762.5969 77.0045
786.976 59.4065
825.4442 55.9814
873.2967 105.5812
965.3122 90.0476
1614.9516 143.2081
1620.8865 109.0718
1630.8883 56.397
1632.4273 38.4934
1636.2663 45.0287
1639.8538 57.4548
1648.438 12.8875
1662.1477 4.4303
1671.4435 3.807
3223.7228 2135.1267
3426.1945 1497.0812
3426.8706 190.4721
3488.4427 170.3801
3495.6916 801.6778
3529.5272 655.9651
3548.4486 420.7448
3794.3813 29.7715
3794.6908 22.9312
3803.3009 95.4889
3806.5919 76.6384
3816.5062 88.9867
3820.5609 85.5639
3833.6755 86.8808
3839.091 73.0826
3869.5407 97.3832
3897.5444 113.8056
3898.5992 108.6707

IXd
6.6348 1.2591
8.1426 3.7613
14.0996 0.18
22.0189 0.5167
28.135 1.7562
44.6864 0.4359
52.4055 0.3897
67.2414 1.7871

71.3035 1.6385
92.2583 25.7711
93.0229 2.3039
97.6612 0.2276
127.8764 18.7938
135.2916 0.3771
141.2352 0.2876
153.1745 189.0218
173.9481 18.4935
183.747 18.9531
187.9323 18.8379
198.0679 68.6278
208.4011 21.0642
216.5884 2.0188
224.8042 61.9663
229.5982 73.1342
246.5581 81.8329
281.3676 17.5065
283.0281 5.2766
290.4259 1.4708
292.6681 10.4208
303.5133 1.9762
312.1249 312.6503
338.7002 6.1543
352.2876 4.0395
357.1059 56.7638
369.1106 13.0189
382.445 1.0519
406.2226 172.2244
417.0506 27.5925
492.5709 88.9116
504.2076 10.0245
519.1712 0.6333
548.5906 103.4849
577.3331 40.1171
629.5416 119.8305
639.5085 43.8045
711.977 29.2502
720.4991 103.779
766.0526 190.0874
859.3933 95.7715
867.2392 33.6536
1017.6826 62.1029
1611.9626 207.2904
1615.6188 40.8822
1620.8245 125.2597
1621.1879 13.332
1631.5982 25.9828
1639.9855 10.6868
1644.6798 37.1668
1659.8477 15.6545
1676.9055 14.1359
3114.4327 2663.3283
3366.4128 991.4559
3393.0332 222.473
3464.7833 916.4085
3561.7631 570.9486
3609.0673 96.9099
3631.4301 419.3807
3761.1439 8.3678
3795.5177 21.0491
3809.1602 73.8721
3809.9341 26.3284
3810.3827 38.9674
3813.4781 284.3108
3838.0389 62.2326
3857.3611 107.4018
3868.7152 95.0526
3869.4239 80.2585
3900.174 105.5592

IXe
7.8427 0.3184
11.2039 4.7738
18.4336 0.7638
25.2449 0.067
38.354 2.9262
44.0416 0.7303
50.7503 7.3814
67.4715 3.7075
71.346 0.1901
93.1489 1.0191
96.8881 1.7887
97.6945 0.4618
109.03 18.6088
128.6501 29.1325
136.1551 0.3767
138.4945 2.9123
152.8207 206.4374
157.9392 11.7404
188.9805 16.0699
190.3701 3.1065
201.0174 178.3619
209.3985 37.4358
216.1208 8.4889
220.2664 26.3181
221.7762 25.4257
226.1247 8.0561
248.1831 54.616
283.8514 1.1663
291.0575 3.6115
293.4204 5.523
302.8811 1.6855
315.4932 331.7789
342.3476 6.413
353.6024 2.9273
357.7425 61.6028
381.3263 16.6414
385.8865 1.2526
405.8065 177.0941
505.4707 89.7732
518.1906 0.0113
548.5929 101.9854
568.7787 1.8373
590.6772 53.614
630.8599 114.0651
646.0491 59.0765
712.6485 56.5162
713.2234 154.2755
718.7031 6.6954
781.5171 177.4947
883.5561 37.6449
1061.6272 63.7657
1608.5599 173.8669
1615.6621 40.2375
1620.8148 122.9043
1621.1623 53.2653
1628.2748 4.015
1632.9616 22.7352
1644.8513 35.7355
1657.3513 27.3343
1677.1959 13.9127
3016.0059 2637.8901
3366.1455 213.4837
3442.1304 1051.549
3584.1197 309.1786
3610.1513 93.1303
3632.0852 406.5637
3645.0783 936.1729
3761.0347 7.6651
3794.3578 10.1611
3794.5885 24.4648
3809.253 95.6201
3809.851 0.41
3810.5448 25.7147
3813.1916 292.9822
3840.1475 57.3187
3857.415 106.9022
3898.3431 1.7037
3898.6311 194.7383

iIXa
25.4741 1.3927
36.6057 2.5332
43.7317 9.5353
49.2073 5.6447
68.011 1.4701
80.681 3.9127
88.9447 3.5845
97.4103 1.367
135.3742 1.2766
138.7724 2.9498
143.8693 4.5861
166.7086 30.2206
172.3008 54.4678
175.2864 21.255
183.9079 43.1574
187.0127 9.0996
208.7365 17.8872
240.4196 2.8857
255.3318 5.8286
261.0143 93.8797
274.67 37.8965
280.5248 29.7164
299.0952 124.0856
302.3669 45.0583
312.6251 30.7968
340.4093 4.9805
346.8632 43.8967
360.4199 87.6941
369.8294 51.0024
411.9783 120.8852
424.3167 106.5023
442.381 12.4506
446.0306 20.0105
485.5772 46.3073
491.2602 35.3606
538.8846 18.8596
572.0154 56.6749
580.8358 34.7189
584.6456 87.1535
604.6254 102.1027
645.9719 73.3024
673.9197 67.6895
719.4835 181.6613
754.2417 214.3358
756.8438 173.9384
781.2105 233.2601
813.2899 52.5595
839.2723 311.6508
884.9387 88.1048
942.3869 92.2294
957.8868 35.1558
967.286 92.8451
1599.4134 6.3549
1602.8262 169.6697
1617.964 131.2177
1625.9085 119.8557
1638.0392 8.968
1657.2792 55.3216
1665.1065 41.0007
1689.9256 14.5621
1733.3738 248.471
3199.0779 1185.0488
3268.1719 1259.0745
3292.1273 575.8078
3309.858 583.0473
3452.8206 1030.7222
3536.471 768.2934
3560.2135 330.1918
3619.2263 696.8133
3705.5962 537.8348
3768.5673 5.0225
3769.8572 20.0557
3803.1112 71.2391
3847.8435 119.5926
3850.7448 100.0653
3860.2611 130.3052
3869.0136 134.4347
3878.2919 132.0258

iIXb
19.5106 0.2138
25.4799 0.7214
31.9917 4.6079
34.4439 5.0969
52.553 2.7505
57.0336 1.6432
66.6445 2.9577
73.7043 25.6619
83.6991 1.3159
104.1766 0.4427
116.1209 19.3232
134.8151 2.7378
148.518 10.6122
158.6742 179.7082
166.7973 18.3403
170.4697 15.8893
176.4322 44.6724
187.6952 28.7961
206.9092 92.7976
250.5826 71.5587
261.6098 49.2906
272.2916 12.2802
284.2793 134.9339
290.2645 147.5529
299.0279 24.4827
307.4885 131.0009
319.1127 61.7919
333.5772 8.7942
350.1643 73.0887
367.6619 23.461
383.7962 28.7089
408.6377 91.195
424.5296 59.6774
440.0579 14.6437
460.2586 14.4849
479.6933 50.8989
499.6063 67.9048
527.5176 73.0064
550.3767 67.7846
585.0004 107.7581
651.5854 31.0827
661.7341 12.3322
685.9023 131.9837
718.3344 51.628
784.1434 325.4133
793.0604 180.2545
812.1755 191.3955
849.7964 42.7827
906.8153 98.2626
939.7665 42.6865
980.6648 129.5468
1136.1717 113.0474
1594.294 91.5392
1596.9148 81.9537
1607.6347 92.5421
1617.6741 98.7753
1629.2053 36.4745
1642.7766 44.8304
1659.6048 64.9255
1671.1362 24.935
1712.9636 226.8636
2937.8275 1329.0213
3212.5752 1426.7942
3315.4562 880.8381
3361.1379 2068.4738
3423.6504 398.214
3499.4526 608.4052
3600.3364 676.4816

3743.5473 68.0655
3777.0371 18.3919
3793.1639 31.7165
3799.8758 71.451
3801.5187 94.4053
3834.4193 94.8411
3859.1626 132.0908
3875.2661 174.2356
3881.7927 117.4479
3895.0118 123.0826

iIXc
15.5993 0.8354
32.2543 3.5409
36.8766 7.0666
62.7614 2.9704
64.4459 2.7554
79.1366 1.7139
89.7524 2.5387
100.2332 1.3216
103.5226 12.3052
123.3336 1.7335
131.4356 6.582
158.9381 2.3299
169.1537 88.6118
176.1571 124.3241
179.7616 37.3284
193.0575 10.54
194.6162 29.8574
209.8087 25.7276
223.9447 52.7704
242.6023 3.4653
245.6473 24.9729
255.8696 21.5625
291.7814 25.6121
296.3026 17.1615
299.4142 5.5256
313.8288 4.9277
330.9612 5.4924
343.2446 65.8191
345.4523 117.5368
368.9581 15.7106
381.4508 103.1791
394.2581 72.8309
398.6181 54.6483
404.7947 89.083
448.8654 7.9331
487.3167 118.0042
499.3735 9.6935
516.2243 45.6964
574.7951 296.4653
592.2338 17.4415
614.9728 45.9372
638.9963 9.9288
661.0315 318.7152
671.4873 67.2128
703.4512 244.4437
784.0906 130.9433
789.1428 66.8161
820.2021 184.4066
833.5238 197.4851
874.8783 80.288
901.7591 36.1297
924.8345 55.6676
1600.2766 168.3843
1603.6689 136.3501
1611.111 103.7005
1612.0876 135.0783
1624.227 47.6153
1639.7027 10.9218
1650.6897 2.1049
1663.9676 29.0241
1692.1166 315.502
3307.4312 476.897
3397.4047 1876.6375
3448.3636 180.1512
3471.217 323.1023
3504.0132 559.6982
3557.0345 630.4728
3592.6028 696.8405
3775.4941 126.1278
3790.2076 103.4586
3792.8874 30.6823
3829.7186 127.4461
3831.1758 123.0399
3835.7556 106.8918
3853.3313 135.1648
3860.823 118.5721
3878.7316 114.1407
3894.5676 119.4583

iIXd
18.0069 0.1814
28.9941 1.8145
30.62 2.7614
41.1688 2.8436
48.9709 1.6393
53.0954 20.6003
68.9524 4.5255
83.2134 6.8549
88.3547 1.6379
97.4964 5.2909
110.0051 3.4128
135.2716 0.5662
155.0785 10.148
166.9174 5.01
170.8903 6.1408
179.7839 182.1184
186.1855 12.5111
201.9575 142.1478
231.002 12.0922
243.3648 60.6955
249.1366 10.7221
268.1305 56.3427
291.8713 19.4532
299.068 54.4496
301.047 5.6623
305.1563 69.901
317.4949 108.0127
329.9075 14.9188
359.6595 54.0214
367.1216 18.4884
399.5243 185.5028
413.6333 122.3278
415.3598 24.8692
422.2557 16.7512
442.654 47.5903
466.7173 11.2828
492.2209 39.767
544.8787 62.899
556.2673 61.3921
610.098 72.6231
626.5909 86.2622
658.8204 87.1645
682.3856 86.0352
739.7253 114.2386
750.9233 229.2145
775.3436 315.0936
810.6934 157.1922
860.8663 161.7213
874.6395 80.8861
919.6556 59.5009
959.3995 70.1435
1024.4497 87.0424
1590.8406 23.6354
1595.7277 117.2087
1603.9684 162.7563
1622.8038 85.0242
1634.4239 45.8721
1649.0191 33.2098

1654.0955	52.7678
1672.0683	30.2619
1715.8996	237.0013
3088.182	1206.1263
3300.6587	1479.489
3308.8773	1145.6988
3403.143	648.5808
3522.1196	359.7644
3540.2118	1065.2664
3639.9863	529.4977
3738.8384	487.6465
3746.4269	36.4766
3772.3159	3.1161
3791.9573	31.0955
3801.3529	65.7241
3835.5317	96.9858
3856.1291	122.8493
3863.2867	128.575
3870.1205	142.213
3893.3371	121.4634
